# Supplementary material for: Optimising the secondary use of primary care prescribing data to improve quality of care: a qualitative analysis
Source: BMJ Open. 2022 Jul 21;12(7):e062349. doi: 10.1136/bmjopen-2022-062349 (PMC9310167; doi:10.1136/bmjopen-2022-062349)
Supplement: Supplementary data [file bmjopen-2022-062349supp001.pdf]

Optimizing the secondary use of primary care prescribing data to improve quality of care: a qualitative analysis

Supplementary file 1: Interview topic guide

1. Purpose of use of primary care prescribing data

- How does your organization currently use primary care prescribing data? Refer to the table on the following page (Supplementary file 2) listing core actors and purposes of use identified. Is this accurate and complete?

- How would you describe the information you need to carry-out your organization's role? (e.g. multi-year information on performance at national-level; aggregate, comparative performance measures on providers; timely, continuous information at patient-level, etc.).

- What actors do you work with directly in the scope of primary care prescribing data?

2. Current use of prescribing data

- **Use of indicators**. Does your organization actively collect data related to the following: (1) Antibiotics; (2) Opioids; (3) Benzodiazepines. If so, what are the indicators or measures used related to each? How long have these been reported on? Is it intended for internal or external use? Who is the target audience (intended user) of the information generated?

- **Data sources**. What is your primary source of primary care prescribing data? (e.g. medical records, administrative data, specific research database, others). How is the data collected? If other actors are involved, whom does this include? Is the data considered of quality?

- **Analysis.** How is the data currently analyzed – benchmarking, time trend, international comparison? How would you describe this analysis? (e.g. time interval, comparators used, aggregation as composite scores, etc.)

- **Dissemination**. How is the data disseminated? What is the format of reporting (print, electronic, web-based)? What is the lag time in presenting analyzed data? How does it reach the intended target audience?

3. Perceived actionability

- In your opinion, how can the process in which data is analyzed and reported on be improved upon?

- Is the information generated useful for your purposes? That is, are you able to make decisions and learn from the information?

- In general, what are the obstacles to the optimal use of primary care prescribing data at present?

Barbazza E, *et al. BMJ Open* 2022; 12:e062349. doi: 10.1136/bmjopen-2022-062349

Supplementary file 2: Mapping of stakeholders and uses of prescribing data

Purpose of use[1]	Stakeholders
**Micro-level**	
Individual professional performance	Individual GP and HIS supplier<br>Community pharmacist and HIS supplier
Practice improvement	GP practice/peers using HIS<br>GP practice and insurers<br>GP practice and affiliate research networks<br>Community pharmacy and pharmacy network
Multidisciplinary improvement	Pharmacotherapy audit groups (GPs and pharmacists) (FTOs)
**Meso-level**	
Organization/ networks performance improvement	Care groups and affiliate GP practices (e.g., MCC Omens Care Group, Zorg In Ontwikkeling)
Quality-based financing	Health Insurers (e.g., Zilveren Kruis)
Monitoring	Lareb Side Effects Center<br>Foundation of Pharmaceutical Statistics (SFK)
Professional development	Dutch Institute for Responsible Drug Use (IVM)<br>National Association of GPs (LHV)
Advocacy and standards	Dutch GP Association (NHG)<br>The Royal Dutch Society for the Promotion of Pharmacy (KNMP)<br>Organization for first line care (InEen)<br>Patient Federation Netherlands (Patienten Federatie)
**Macro-level**	
Strategy development	Ministry of Health, Welfare and Sport
System performance	National Institute for Public Health and the Environment (RIVM)
System quality assurance	Medicines Evaluation Board (MEB)<br>National Health Care Institute (ZiNL)<br>Health Care Inspectorate (IGJ)<br>Dutch Healthcare Authority (NZa)
**Cross-cutting**	
	Netherlands Institute for Health Services Research (Nivel)<br>Institute for Drug Outcomes Research (Pharmo)<br>Vektis<br>Nictiz<br>Digitalis

[1]Purposes of use draw from the study findings: Barbazza E, Klazinga NS, Kringos DS. Exploring the actionability of healthcare performance indicators for quality of care: a qualitative analysis of the literature, expert opinion and user experience. BMJ Quality & Safety 2021;30:1010-1020.

Barbazza E, *et al. BMJ Open* 2022; 12:e062349. doi: 10.1136/bmjopen-2022-062349

Supplementary file 3: Characteristics of informants

Table S3.1. Elaborated breakdown of informants and non-participants

Characteristics	Total informants N=28		Non-participants N=25		
	n	%	No reply	Unavailable	Contact mediating
**Healthcare system level (context)**					
Micro (clinical)	1 (4)	4	1	2	1
Meso (organizational)	11	39	6	0	5
Macro (policy)	9	32	3	0	2
Cross-cutting (research, EHR supplier)	7	25	2	1	2
**Type of stakeholder**			**12**	**3**	**10**
Association (patient, professional)	8	29	3	0	2
Care group (network)	2	7	0	1	0
Government health agency	9	32	3	0	2
Health professional	1 (4)	4	0	1	1
EHR supplier	4	14	1	0	2
Insurer	1	4	3	0	3
Research	3	11	2	1	0
**Gender**					
Female	8	29	4	2	4
Male	20	71	8	1	6

EHR: Electronic health record.
[a]Numbers in round brackets indicate the total number of informants when individuals with multiple affiliations are accounted for.

Table S3.2. Overview of informants

#	Code	Level	Stakeholder	Gender	Format
1	Association–1	Meso	The Royal Dutch Society for the Promotion of Pharmacy (KNMP)	Female	Phone
2	Health professional–2	Micro	Health Professional	Male	Phone
3	Care group–3	Meso	MCC Omens Care Group	Female	Phone
4	Care group–4	Meso	ZIO	Female	Phone
5	Government–5	Macro	Medicines Evaluation Board (MEB)	Male	Phone
6	EHR supplier–6	Cross-cutting	Digitalis	Male	In-person
7	Association–7	Meso	Organization of Firstline Care (InEen)	Male	Phone
8	Association–8	Meso	Dutch Institute for Responsible Drug Use (IVM)	Female	Phone
9	EHR supplier–9	Cross-cutting	Nictiz	Male	Written
10	EHR supplier–10	Cross-cutting	CampuGroup Medical (CGM)	Male	Phone
11	Government–11	Macro	Ministry of Health, Welfare and Sport	Male	Phone
12	Association–12	Meso	Lareb Side Effects Center	Male	Phone
13	Association–13	Meso	Foundation for Quality Indicators Pharmacy (SFK)	Male	Phone

BMJ Publishing Group Limited (BMJ) disclaims all liability and responsibility arising from any reliance placed on this supplemental material which has been supplied by the author(s)

#	Code	Level	Stakeholder	Gender	Format
14	Government–14	Macro	National Institute for Public Health and the Environment (RIVM)	Male	Written
15	Association–15	Meso	Dutch General Practitioners Association (NHG)	Male	Phone
16	Government–16	Macro	Health Care Inspectorate	Male	Phone
17	Research–17	Cross-cutting	Institute for Drug Outcomes Research (Pharmo)	Male	Phone
18	Association–18	Meso	Patient Federation	Male	Phone
19	Insurer–19	Meso	Zilveren Kruis	Male	Phone
20	Association–20	Meso	National General Practitioners Association (LHV)	Male	Phone
21	EHR supplier–21	Cross-cutting	Vektis	Male	Phone
22	Government agency–22	Macro	National Health Care Institute	Male	Phone
23	Government agency–23	Macro	Ministry of Health, Welfare and Sport	Male	Phone
24	Government agency–24	Macro	Dutch Healthcare Authority	Female	Phone
25	Government agency–24	Macro	Dutch Healthcare Authority	Male	Phone
26	Research–25	Cross-cutting	Netherlands Institute for Health Services Research	Female	In-person
27	Research–25	Cross-cutting	Netherlands Institute for Health Services Research	Female	In-person
28	Government agency–26	Macro	National Institute for Public Health and the Environment (RIVM)	Female	Phone

EHR: Electronic health record.

Barbazza E*, et al. BMJ Open* 2022; 12:e062349. doi: 10.1136/bmjopen-2022-062349
