# Supplementary material for: Optimising the secondary use of primary care prescribing data to improve quality of care: a qualitative analysis
Source: BMJ Open. 2022 Jul 21;12(7):e062349. doi: 10.1136/bmjopen-2022-062349 (PMC9310167; doi:10.1136/bmjopen-2022-062349)
Supplement: Author's manuscript [file bmjopen-2022-062349.draft_revisions.pdf]

BMJ Open

BMJ Open is committed to open peer review. As part of this commitment we make the peer review history of every article we publish publicly available.

When an article is published we post the peer reviewers' comments and the authors' responses online. We also post the versions of the paper that were used during peer review. These are the versions that the peer review comments apply to.

The versions of the paper that follow are the versions that were submitted during the peer review process. They are not the versions of record or the final published versions. They should not be cited or distributed as the published version of this manuscript.

BMJ Open is an open access journal and the full, final, typeset and author-corrected version of record of the manuscript is available on our site with no access controls, subscription charges or pay-per-view fees (http://bmjopen.bmj.com).

If you have any questions on BMJ Open's open peer review process please email info.bmjopen@bmj.com

BMJ Open

Optimizing the secondary use of primary care prescribing data to improve quality of care: a qualitative analysis

Journal:	*BMJ Open*
Manuscript ID	bmjopen-2022-062349
Article Type:	Original research
Date Submitted by the Author:	01-Mar-2022

Complete List of Authors:	Barbazza, Erica; Amsterdam UMC Locatie AMC, Department of Public and Occupational Health<br>Verheij, Robert A; Netherlands Institute for Health Services Research, Learning Health Systems Research Programme<br>Ramerman, Lotte; Netherlands Institute for Health Services Research, Learning Health Systems Research Programme<br>Klazinga, Niek; Amsterdam UMC Locatie AMC, Department of Public and Occupational Health<br>Kringos, D; Amsterdam UMC Location AMC, Department of Public and Occupational Health

Keywords:	PRIMARY CARE, Quality in health care < HEALTH SERVICES ADMINISTRATION & MANAGEMENT, QUALITATIVE RESEARCH, Health & safety < HEALTH SERVICES ADMINISTRATION & MANAGEMENT, Information management < BIOTECHNOLOGY & BIOINFORMATICS

SCHOLARONE™
Manuscripts

I, the Submitting Author has the right to grant and does grant on behalf of all authors of the Work (as defined in the below author licence), an exclusive licence and/or a non-exclusive licence for contributions from authors who are: i) UK Crown employees; ii) where BMJ has agreed a CC-BY licence shall apply, and/or iii) in accordance with the terms applicable for US Federal Government officers or employees acting as part of their official duties; on a worldwide, perpetual, irrevocable, royalty-free basis to BMJ Publishing Group Ltd ("BMJ") its licensees and where the relevant Journal is co-owned by BMJ to the co-owners of the Journal, to publish the Work in this journal and any other BMJ products and to exploit all rights, as set out in our licence.

The Submitting Author accepts and understands that any supply made under these terms is made by BMJ to the Submitting Author unless you are acting as an employee on behalf of your employer or a postgraduate student of an affiliated institution which is paying any applicable article publishing charge ("APC") for Open Access articles. Where the Submitting Author wishes to make the Work available on an Open Access basis (and intends to pay the relevant APC), the terms of reuse of such Open Access shall be governed by a Creative Commons licence – details of these licences and which Creative Commons licence will apply to this Work are set out in our licence referred to above.

Other than as permitted in any relevant BMJ Author's Self Archiving Policies, I confirm this Work has not been accepted for publication elsewhere, is not being considered for publication elsewhere and does not duplicate material already published. I confirm all authors consent to publication of this Work and authorise the granting of this licence.

For peer review only

Original research

Optimizing the secondary use of primary care prescribing data to improve quality of care: a qualitative analysis

Authors
Erica Barbazza MSc[1], https://orcid.org/0000-0001-7621-1638
Robert Verheij MSc PhD[2], https://orcid.org/0000-0003-2234-4819
Lotte Ramerman MSc PhD[2], https://orcid.org/0000-0001-8436-0924
Niek Klazinga MD PhD[1], https://orcid.org/0000-0002-3937-8014
Dionne Kringos MSc PhD[1], https://orcid.org/0000-0003-2711-4713

[1]Department of Public and Occupational Health, Amsterdam UMC, University of Amsterdam, Amsterdam Public Health research institute, Amsterdam, the Netherlands
[2]Learning Health Systems Research Programme, Netherlands Institute for Health Services Research, Utrecht, the Netherlands

Address for correspondence
Erica Barbazza
Department of Public and Occupational Health
Amsterdam UMC, Academic Medical Centre
University of Amsterdam
Meibergdreef 9, 1105 AZ, Amsterdam, the Netherlands
Email: e.s.barbazza@amsterdamumc.nl
Tel.: +45 31 12 17 11

Word count abstract: 298 words

Word count main text: 4,694 (excluding figures, tables, references)

Figures and tables: up to 5

Supplementary material
COREQ checklist
Supplementary file 1: Interview topic guide
Supplementary file 2: Mapping of stakeholders and uses of prescribing data
Supplementary file 3: Characteristics of informants

Running title
Primary care prescribing data in the Netherlands

Keywords
primary care; prescriptions; quality indicators; performance measures; the Netherlands; health information management

For peer review only - http://bmjopen.bmj.com/site/about/guidelines.xhtml

Abstract

Objectives. To explore available data sources, secondary uses, and key considerations for optimizing the actionability of primary care prescribing data to improve quality of care in the Dutch context.

Design. An exploratory qualitative study was undertaken based on semi-structured interviews. We anchored our investigation around three tracer prescription types: antibiotics; benzodiazepines; and opioids. Descriptive and explanatory themes were derived from interview data using thematic analysis.

Setting. Stakeholders were sampled from across the micro (clinical), meso (organizational), and macro (policy) contexts of the Dutch primary care system.

Participants. The study involved 28 informants representing general practitioners (GPs), community pharmacists, regional chronic care networks (care groups), academia and research institutes, insurers, professional associations, electronic health record (EHR) vendors, and national authorities.

Results. In the Netherlands, three main sources of data for improving prescribing in primary care are in use: clinical data in the EHRs of GP practices; pharmacy data in community pharmacy databases; and claims data of insurers. While the secondary use of pharmacy and claims data is well-established across levels, the use of these data together with EHR data is limited. Important differences in the types of prescribing information needed by micro-meso-macro context are found, though the extent to which current indicators address these varies by prescription type. Five main themes were identified as areas for optimizing data use: (1) measuring what matters, (2) increasing data linkages, (3) improving data quality, (4) facilitating data sharing, and (5) optimizing fit for use analysis.

Conclusions. To make primary care prescribing data useful for improving quality, consolidated patient-specific data on the indication for a prescription and dispensed medicine, over time, is needed. In the Netherlands, the selection of indicators requires further prioritization to better signal the appropriateness and long-term use of prescription drugs. Prioritizing data linkages is critical towards more actionable use.

For peer review only - http://bmjopen.bmj.com/site/about/guidelines.xhtml

Strengths and limitations of this study

- Semi-structured interviews elicited firsthand insights into the secondary use of primary care prescribing data, filling this knowledge gap in the published literature.
- Stakeholder interviews spanned all levels of the Dutch healthcare system and engaged varied perspectives, including community pharmacists and general practice, offering diverse insights.
- Three tracer prescription types were selected to anchor discussions with stakeholders and the findings may not capture the nuances of all prescriptions.
- Our study is deliberatively exploratory in nature, thus patterns and experiences by stakeholder types require testing with a larger sample, including patients, before they can be generalized.

For peer review only

For peer review only - http://bmjopen.bmj.com/site/about/guidelines.xhtml

Introduction

Improving prescribing practices has received increasing policy attention globally. This prioritization follows concerning trends, including rising levels of antimicrobial resistance [1,2], an epidemic of opioid use [3-5], and the increasing misuse of benzodiazepines [6-8]. In the Dutch context—like other gatekeeping models of primary care—general practitioners (GPs) function as the first-line for patient management and entry-point to secondary healthcare services. In effect, GPs together with community-based pharmacists are central to services including the issuing and refilling of outpatient prescription medicines [9]. Measuring the performance of services provided by GPs and community pharmacists (both key primary care providers) is fundamental to improve quality [10]. Hence, the use of quality indicators, as a measurement tool to quantify quality, is of critical importance [11-13].

In the Netherlands, the far-reaching digitalization of patient data and physician prescribing has long been recognized as a powerful resource for improving quality [14-16]. All GP practices (approximately 5,000) record data in electronic health records (EHRs) supplied by ten main EHR vendor brands on the market [16]. Since 2014, primary care prescriptions are issued electronically for dispensing medicines at one of approximately 2,000 community pharmacies across the country [16]. The resulting electronic primary care prescribing data has secondary uses that extend across the micro (clinical care), meso (organizations and networks), and macro (policy) context of the Dutch healthcare system [17].

However, as health services research has increasingly called attention to, the availability of data alone does not guarantee its *use* for quality-related decision-making [18,19]. The information produced should also be actionable [20]. The movement towards learning healthcare systems further attests to the critical role of actionable data as an integral part of healthcare delivery processes [21,22]. In primary care, given the critical potential of prescription data to indicate, for instance, inappropriate prescriptions, overprescribing, addiction issues, or antimicrobial resistance trends, it is essential to ensure healthcare systems are optimally using available prescription data for learning and decision-making purposes towards quality improvement in practice.

In the data-rich context of the Netherlands, activity around the use of healthcare data is high: survey data finds Dutch GPs regularly receive as many as 10 different feedback reports [23]. This volume of activity has called into question the extent to which performance indicators are actually *used* for improvement purposes. Research on the secondary uses of healthcare data has been conducted in the context of Dutch hospitals [24], out-of-hours care [25] and integrated care networks [26]. In the absence of an overview of routine primary care prescribing data sources, what and how available data is used to improve quality across the healthcare system is unclear.

In this study, we set out to investigate through the firsthand insights of stakeholders of the Dutch healthcare system, secondary uses of primary care prescribing data for quality of care. We also aimed to distill insights into opportunities to improve the use of prescribing data for quality-related decision-making. To anchor our investigation and generate concrete, practical examples of prescribing data uses, we focused on three commonly prescribed types of prescriptions: antibiotics; benzodiazepines; and opioids. The prescriptions are each of significant societal and public health importance [27,28] and vary in their etiological and therapeutic use (infection control, psychological disorders, and pain management, respectively). In combination, the selected prescription types can offer insights into the use of primary care prescribing data as a whole.

With this aim and focus, the study is guided by the following three questions: what are the available sources and characteristics of primary care prescribing data? How is this data currently used for improving quality of care? And, what are key considerations for optimizing the secondary uses of primary care prescribing data?

For peer review only - http://bmjopen.bmj.com/site/about/guidelines.xhtml

Methods

Design

An exploratory qualitative study design was employed [29]. Reporting adheres to the Consolidated Criteria for Reporting Qualitative Research [30]. Semi-structured interviews with stakeholders ranging the clinical (micro), organizational (meso) and policy (macro) context of the Dutch healthcare system were conducted for rich individual exchanges and practical, system-spanning insights [31]. The research team included experts on healthcare performance intelligence, primary care, health information systems, and the Dutch context. The primary researcher and interviewer is an experienced qualitative researcher and doctoral student on the actionability of healthcare performance indicators.

To operationalize the construct of actionable indicators, we drew from an existing definition depicting actionability as the two related constructs of *fitness for purpose*—information serving an intended decision-making function—and *fitness for use*—the ability to get the right information, into the right hands at the right time [20]. To explore fitness for purpose, the definition's differentiation of types of uses of indicators across healthcare systems was applied. This depiction of actionable indicators, together with our three main research questions, served as the framework for our interview guide. Specifically, the themes explored with informants included: sources of primary care prescribing data; current uses of prescribing data (anchored in the selected prescription types); and perceived actionability constraints (Supplementary file 1).

Sample and recruitment selection

We defined our target informants by Dutch stakeholders across the micro-meso-macro contexts of the healthcare system with firsthand use of primary care prescribing data for monitoring, assessing and/or improving quality. We identified more than 20 different stakeholders (Supplementary file 2). An initial listing was prepared based on reviews of key literature [16,32,33] and the expertise of the study team. The list was validated with an existing Dutch network (Data Expert Community), with representation of national stakeholders working in the field of healthcare data. Feedback from the network was solicited at an in-person meeting in November 2019 in Utrecht, the Netherlands.

We used multiple methods to reach prospective informants affiliated to the stakeholders identified. First, we reviewed the webpages of target stakeholders for contacts and membership lists. Second, the authorship of literature related to primary care and medicines in the Dutch context (e.g., scientific articles, reports, evaluations, factsheets, presentations) was extracted. Third, the expertise of the study team and advice of external experts, as well as prospective informants, was solicited, and a snowballing approach was applied. Prospective informants were invited to participate in the study via email by the authors (EB,RV,LR) and received an overview document detailing the background, aim, scope and research questions.

Data collection

Interviews were conducted over a four-month period (November 2019–February 2020). Interviews ranged 30–60 minutes in length. They were conducted both in-person and at-distance by phone, based on the proximity and preference of informants. In instances where informants requested to extend an invitation to colleagues, these interviews were conducted jointly. We also accommodated requests to answer questions in writing. With the agreement of informants, interviews were recorded and transcribed verbatim. Regular meetings with the full study team were organized to exchange on the process and emerging themes. The interviews were considered complete when the range of informants represented stakeholders spanning the micro-meso-macro levels of the healthcare system.

Data analysis

Thematic analysis was used to analyze interview data [34] in an Excel tool developed in the approach of Meyer and Avery [35]. The analysis process included familiarization with the data, development of a coding framework, coding, mapping and interpretation of results. The coding framework was developed based on the items of the semi-structured interviews: purposes of use; actors; indicators; data sources; analysis; dissemination; barriers; and opportunities for improvement (Supplementary file 2).

For peer review only - http://bmjopen.bmj.com/site/about/guidelines.xhtml

Additional themes were generated through open (unrestricted) coding in an inductive approach. The initial coding and clustering of themes was conducted by the primary researcher. To ensure validity of the findings, the results were regularly reviewed by the full study team. In reporting on the results by research question, verbatim quotes were extracted from the transcripts.

Ethics

The research protocol was developed in accordance with the ethical requirements of the primary research affiliation to Amsterdam University Medical Centers of the University of Amsterdam and relevant Dutch ethics guidelines [36]. To ensure informed voluntary participation, informants contributing to this study provided written informed consent to participate during the recruitment stage and restated their consent verbally at the start of interviews. All interview data has been anonymized. Confidentiality was assured by referring to informants by stakeholder type and an assigned number (e.g., Health professional–1).

Patient and public involvement

The preliminary findings were shared at an international scientific conference in 2021. The interaction with participants provided a unique opportunity for critically reflecting on the findings. A preprint version of the study will be circulated to interviewees, and once published, the results will be reported back to all.

For peer review only - http://bmjopen.bmj.com/site/about/guidelines.xhtml

Results

Characteristics of informants

In total, 53 informants were contacted of which 28 were interviewed representing 26 different stakeholders. Non-participants were either unresponsive (n=12), referred to an alternative contact (n=10), or unavailable due to time constraints (n=3). Two interviews were conducted with two informants present. In two other instances, information was collected via email exchange only, at the preference of the informant. No repeat interviews were carried out. Some informants held multiple affiliations. Notably, three informants were both health professionals and affiliated to another stakeholder. For the purposes of reporting, only one primary affiliation has been used (Table 1). See Supplementary file 3 for a detailed overview of informant characteristics.

Table 1. Summary of informant characteristics

Characteristics	Total informants N=28	
	n	%
**Healthcare system level (context)**		
Micro (clinical)	1	4
Meso (organizational)	11	39
Macro (policy)	9	32
Cross-cutting (research, EHR supplier)	7	25
**Type of stakeholder**		
Association (patient, professional)	8	29
Care group (network)	2	7
Government health agency	9	32
Health professional	1	4
EHR supplier	4	14
Insurer	1	4
Research	3	11
**Gender**		
Female	8	29
Male	20	71

EHR: Electronic health record.

Sources and characteristics of primary care prescribing data

Three main sources of primary care prescribing data for secondary uses towards improving quality are in use in the Netherlands: clinical data in the EHRs of GP practices and dispensing data related to prescriptions dispensed in community pharmacy databases and claims for prescriptions of insurers.

Datasets which can be combined and supplemented with other information are available, specifically: the Institute for Drug Outcomes Research Database [37], Nivel Primary Care Database [15,32,38] and various research-specific datasets of academic networks of GPs (e.g., Registration Network Groningen [39]). These datasets have the advantage of more complete information (diagnosis and dispensed medicines) though are limited to the voluntary participation GP practices. Other types of prescribing data though not specific to primary care include self- or physician-reported medicines' side effects [40] and in-patient prescribing in hospital databases.

Table 2 summarizes these data sources, the nature of information and advantages, and limitations of each for secondary quality-related uses as described by informants. According to informants, not one data source is considered *complete*, as each has unique advantages, but also limitations as a potential source for quality-related decision-making. Clinical data in EHRs captures the diagnosis (indication) for a prescription, though depending on the EHR system can lack details on the medicines retrieved and

For peer review only - http://bmjopen.bmj.com/site/about/guidelines.xhtml

dispensed in community pharmacies. Conversely, administrative pharmacy data and insurance claims are rich in details of prescriptions dispensed and reimbursed, though lack clinical details found in EHRs, specifically associated laboratory results and a specific diagnosis. As informants described:

> The missed link between the diagnosis in the EHR and what is dispensed as the medication, leaves little insights into whether the prescription provided was the right one or necessary. (Health professional–2)

> From the pharmacist perspective, the absence of a link to a specific diagnosis means that interpreting values requires in most instances more analysis and reflection. (Association–13)

For peer review only

For peer review only - http://bmjopen.bmj.com/site/about/guidelines.xhtml

Table 2. Primary care prescribing data landscape in the Netherlands according to informants

Data source	Repository	Coverage	Nature of information	Advantages	Limitations
Clinical	EHRs	All GP practices	Prescription level data with patient ids including complete medical history, diagnosis, lab tests and prescribed medicines.	Includes indication for prescription. Possibility to link across databases using unique patient identifier. Possible to link with comorbidities.	Lacks data on prescriptions filled and dispensed by pharmacist. No central database. Varied recording of data across EHR suppliers.
Pharmacy dispensing data of community pharmacist	Foundation of Pharmaceutical Statistics	Across community pharmacies	Patient-level information on dispensed medicines in pharmacy system, medication including type, dosage, other medications.	Complete overview of dispensed medicines by community pharmacies.	Lacks data on diagnosis and lab results. Excludes: prescriptions issued but not retrieved; over-the-counter medicines; prescriptions issued and dispensed in hospitals.
Claims (pharmacy, services)	Drug Information Project (Dutch Health Care Institute)	Across community pharmacies	Information on prescription (e.g., dosage, quantity dispensed), prescriber, dispensing pharmacy and price declared/ reimbursed filled by public pharmacies.	Data collected across all practices/public pharmacies.	Lacks data on diagnosis. Includes data only for reimbursed medicines and services.
Other reposit-ories	Nivel Primary Care Database (Nivel)	Affiliate GP practices from across the country[1]	Data on consultations, diagnosis, prescribed medicines, with the possibility to link other data sources for environmental characteristics, migration background, income, insurance claims, pharmacy data.	Possibility to combine and supplement EHR data with information about pharmaceutical care and secondary level care.	EHR data from affiliated practices only, though representation across the country (10% of the population).
	Pharmo Data Network (Pharmo)	Affiliate care groups[2]	Linked data from public pharmacy database, GP database, hospital pharmacy databases, clinical laboratories.	Possibility to link to EHR data to administrative insurance claims data and pharmacy data.	Data from affiliate care groups only.
	Academic GP network databases	Networks in catchment area of large university hospitals	Patient-level data including complete medical history, diagnosis, medications, etc. for affiliated practices.	Includes indication for prescription. Possibility to link across databases using unique patient identifier.	Limited to affiliate GP practices. Research-specific uses of data.
	Vektis database (Vektis)	Across health care insurers	Insurers claims database of all reimbursed services with data on physician services (e.g., reason for visit) and procedures (e.g., tests).	Completeness of database, with data spanning across the Dutch population and insurers.	Lacks data on diagnosis. Includes data only for reimbursed medicines and services.

Notes: EHR=electronic health record; GP=general practitioner; Nivel=Netherlands Institute for Health Services Research; Pharmo= Institute for Drug Outcomes Research Database. [1]Approximately 500 GP practices, 1.7 million patients; [2]Approximately 13 care groups, 4 million patients.

For peer review only - http://bmjopen.bmj.com/site/about/guidelines.xhtml

Secondary uses of primary care prescribing data

The secondary uses and sources of primary care prescribing data are summarized to follow. See Supplementary file 1 for a detailed table. These descriptions are anchored in the illustrative prescription types applied. At the outset, the information needs by decision-making context and prescription type were differentiated by informants (Table 2).

Table 2. Summary of differentiated information needs by type of prescription described by informants

Context	Antibiotics	Benzodiazepines	Opioids
**Macro** (policy)	What is the overall volume of antibiotics prescribed annually?	How many elderly patients have a long-term benzodiazepine prescription?	What is the overall volume of opioids prescribed? How many are chronic opioid users?
**Meso** (organiza-tional)	How does the volume of prescribing compare with previous years? (care groups)	How does the volume of prescribing compare with previous years and age groups?	How does the volume of prescribing compare with previous years and age groups?
**Micro** (clinical)	Have I prescribed antibiotics appropriately for infections?	How many of my patients have a long-term prescription? How many prescriptions were new versus refills?	How many of my patients have a long-term prescription? How many prescriptions were new versus refills?

Micro-level. Claims data of insurers is used to provide quality feedback on prescribing to GPs in a report called 'practice mirrors' introduced in 2018. These feedback reports detail the volume and costs of prescriptions and can signal GPs that overuse or underuse prescription medications. GPs participating to the Nivel, Pharmo or academic GP research network datasets receive additional feedback on their prescribing patterns.

Nearly all GPs in the Netherlands participate in pharmacotherapy audit groups (FTOs). FTOs are organized locally and are a practical mechanism for creating linkages between GPs and the pharmacists. As one informant described:

> From my experience as a GP, the FTO is a great mechanism for linking up the GP and the pharmacists as the pharmacist really is the one that has a lot of data on what medicines are being handed out. The pharmacist has a really powerful dataset but they do miss the facts about the patient's actual needs. The linkage [exchange] between a GP and the pharmacists data set happens only at the meeting [FTO] itself. (Health Professional–2)

Informants described the indicators reported on at the micro-level vary for reasons primarily due to the type of data available to stakeholders, the priorities of practices and the relevance of existing indicators. On the latter, informants noted differences between feedback that may be useful for a pharmacist versus a GP. For example, from the perspective of pharmacists, the following was described regarding benzodiazepines over an extended period of time:

> There are some indicators to give feedback to pharmacists about whether they give long-term prescriptions to elderly people. But we do not use this as a quality indicator because the pharmacist's care is just a small amount of the care that is provided to patients using benzodiazepines…It depends [rather on] the work of the GPs. (Association–1)

In contrast, from the perspective of GPs, informants described structured feedback on antibiotics as limited by gaps in information, such as the absence of data on how long a patient actually took antibiotics.

Meso-level. Two main types of arrangements are in place for providing feedback at the meso-level. These include regional groups, specifically care groups, as geographically defined networks of

For peer review only - http://bmjopen.bmj.com/site/about/guidelines.xhtml

healthcare providers which provide feedback to affiliated practices. Additionally, research and academic GP networks, such as the Nivel primary care database and GP practices organized around academic hospitals, also conduct research on specific indicators of interest to affiliated GPs.

Dutch professional associations for GPs (e.g., National Association of GPs, Dutch GP Association) and for pharmacists (e.g., Royal Dutch Society for the Promotion of Pharmacy) provide feedback on prescribing for professional development purposes. In the sphere of community pharmacy, the number of medication reviews, participation in pharmacotherapy meetings (FTOs), as well as indicators related to dispensing amounts are indicators regularly measured.

Uses of primary care prescribing data for monitoring purposes by meso-level organizations was described by volume indicators related to the total prescriptions annually, compared to previous years and by age groups. Active monitoring of benzodiazepines at the meso-level was noted to have decreased following changes in reimbursement coverage from January 2009. As one informant explained:

> Around three quarters of prescriptions for benzodiazepines are not reimbursed and data [used] relies on the reimbursement claims. (Association–8)

Moreover, as another informant described with regards to monitoring the uses of prescribing data more locally (e.g., by regions), overall activity is currently limited.

> The discussion on the use of prescriptions at the moment is taking place at the national-level and at the local level but not at the regional-level. This may and is likely to change in the coming years as care groups are more actively involved in the regional implementation of policies. (Association–15).

Macro-level. At the macro-level, pharmacy and claims data are used for strategy development, system performance measurement and quality assurance purposes. Indicators related to the tracer prescriptions are also reported for international comparisons (e.g., total volume of antibiotics for systemic use, elderly patients with prescription of long-term benzodiazepines or related drugs and overall volume of opioids prescribed). A number of policy initiatives are in place to monitor antibiotic prescribing and opioids. However, with regards to benzodiazepines, informants described this as a less pertinent priority following the change in reimbursement resulting in an overall decreasing trend in the number of benzodiazepines prescribed.

Optimizing the use of primary care prescribing data

Five main themes were identified as areas for optimizing the use of primary care prescribing data: (1) measuring what matters, (2) increasing data linkages, (3) improving data quality, (4) facilitating data sharing, and (5) optimizing fit for use analysis. Theme one pertains to methodological considerations about the indicators in use, while themes two, three and four relate to contextual considerations, specifically, the underlying information system and regulations. The last theme is found to reflect managerial considerations influencing an indicator's use in practice. The themes are described to follow.

Measuring what matters. "We have the data. We don't have the right indicator" (Health professional–2). Similar statements were made in reference to indicators currently in use, in particular at the micro-level. Specifically, the absence of indicators to monitor the stop-date of prescriptions were noted, despite the relevance of this information to limit over–re-prescriptions. Information on the stop-date for prescriptions was described of growing importance. Notably, as GPs increasingly work in teams and multiple practices, there is greater potential for re-prescribing to go unnoticed. Similarly, the absence of indicators that differentiate between new versus repeat refills, as well as indicators for monitoring "de-prescribing" were noted as an information gap, especially for measuring quality of chronic care services.

The lack of indicators to measure the appropriateness of prescriptions was also raised:

For peer review only - http://bmjopen.bmj.com/site/about/guidelines.xhtml

> Instead of receiving, 'this month you prescribed this many antibiotics' to know 'this month you prescribed this many antibiotics for this many patients diagnosed with infections' can provide more insights into a GP's actual performance. (Association–15)

> Dispensing data we have is really useful for the overall consumption, but it is limited to assess the quality of care. For example, for antibiotics use and to determine the appropriateness of the use you really need to have the diagnosis data. (Association–1)

Increasing data linkages. The interoperability of data systems was a recurrent theme across informants from all levels of the healthcare system. The challenge to link data sources was described both *within* primary care (GPs and community pharmacists) but also *across* levels (GPs, hospitals and community pharmacists). At present, a reliance on manual data exchange between stakeholders was depicted (e.g., patients providing data to community pharmacists following hospital discharge, pharmacists providing data to GPs at FTO meetings). While in part a consequence of privacy regulations, informants underscored issues of fragmentation and siloed data systems.

> In a perfect world we would have more linkages between the GP databases and that of the pharmacy. Because we know that the systems in the GP practice is lacking some of the information that is available to the pharmacist. Also, what is prescribed in hospital. We need a connection between these systems to create really good indicators. (Association–8)

In the absence of data linkages within primary care as well as specialized care, informants emphasized the implications on the completeness of data and potential to "see the whole picture" (EHR supplier–10).

Improving data quality. The quality of coding is a fundamental challenge to the secondary use of prescribing data. As one informant described:

> If a GP wants to prescribe antibiotics, then they can also change the code, for example, if someone presents with a possible infection and I see they are quite sick, I can code this differently. (Association–15)

Additionally, the poor quality of coding itself was raised:

> In many GP practices at the moment there is simply not enough attention for the quality of the prescription [coding]. GPs are using very old codes [medication codes] in their prescriptions, simply by way of copying their old prescriptions. (EHR supplier–10)

The pertinence of this issue is well-studied (e.g., [41]) and is underscored in projects such as Nivel's formulary-oriented prescribing initiative (Formulariumgericht voorschrijven) [42], where attention is called to improving the quality of GP prescribing.

Facilitating data sharing. Informants raised privacy barriers as a key cause for untapped opportunities to stimulate data sharing across the healthcare system. The European General Data Protection Regulation (GDPR) and national privacy and data ownership policies were referenced as challenges to the sharing and connecting of different sources of data. As one informant described: "It is a political issue of clarifying who is in fact the owner of the data" (Association–14). Informants emphasized the importance of addressing privacy constraints and data sharing in order to allow for more extensive uses.

Actionable analysis. Informants across all levels described limitations regarding the usefulness of analyzed data to inform decision-making. Specifically, at the micro-level opportunities to improve the use of comparators were detailed. For example, the current practice of providing an individual GP with feedback on their performance in relation to the national level was described as too aggregate a summary. The consequence, as one informant noted, is a tendency to defer accountability and cite the

For peer review only - http://bmjopen.bmj.com/site/about/guidelines.xhtml

uniqueness of one's practice population as a cause for deviating trends. In another example, an informant described the compromised actionability of feedback:

> Informing 'you are adhering to guidelines in 80% of prescriptions issued' is not helpful to a GP. It leaves unanswered questions, such as, what patients were involved. (Association–8)

Other obstacles described included the ability to discriminate performances to capture practice variation, with one informant stating: "the problem with the analysis is that the results are not wide. Everyone ends up at the same place" (Insurer–19). Additionally, analyzed data fails to capture at-risk patients and vulnerable groups, of relevance across the micro-meso-macro context. As one informant described from the perspective of pharmacists, current indicators and approaches to analyze information are strained to provide clear direction for improvement related to care for patients with greatest needs:

> I think we need more data to better target the patients that are in need of additional care. Not everyone needs additional, specialized care. It's the 20% that needs additional, specialized care, and for that, our pharmaceutical database is not sufficient. (Association–1)

Obstacles to analyze data that meets the timeliness needs of decision-makers were also described as a hurdle to the use of data. One informant detailed this challenge extends to the timeliness and accessibility of the way in which data is ultimately delivered to end-users: "We miss a dashboard or system that would allow gaining access and make use of the available data" (Association–12).

For peer review only - http://bmjopen.bmj.com/site/about/guidelines.xhtml
For peer review only

Discussion

In this study, we set out to investigate sources, secondary uses, and key considerations for optimizing the actionability of primary care prescribing data. Much of the existing literature on measurement for improving primary care prescribing focuses on implementation sciences and practice-level interventions (e.g. [43-45]). We add to this evidence by adopting a healthcare performance intelligence lens and exploring the actual use of primary care prescribing data in the data-rich context of the Dutch healthcare system.

Our study confirms the numerous secondary uses of electronic primary care data across the clinical, organizational and policy context in the Netherlands. Nonetheless, data are constrained by professional and organizational siloes and perceived privacy constraints that compromise the completeness of information for secondary uses. Importantly, resolving data-related barriers alone will not increase the use of prescribing data. Attention to the development and *use* of indicators that meet the needs of decision-makers is needed. These findings are further described to follow.

First, with regards to primary care prescribing data sources, the challenge of incomplete individual data sources, as described by informants, is in part a phenomenon unique to primary care. For contrast, prescribing in secondary care is marked by clear start (admission) and end processes (discharge or death), with the potential to generate detailed inpatient data for the duration of treatment [46]. Primary care prescribing data, however, tends to lack a way of knowing whether medication was collected and consumed. In effect, primary care prescribing data alone is inherently strained to provide a complete picture of a patient's interaction with the healthcare system for the purpose of improving quality [46,47].

Providing a complete snapshot of primary care prescribing is further strained by linkage limitations. The siloing of data by types—clinical, dispensing, insurer—as described by the informants, is consistent with recent reporting on the Dutch health information system in general [17]. This challenge of data fragmentation is common to many European routine healthcare information systems [47-49], despite that this is not necessarily a legal constraint. In fact, others have argued GDPR leaves much room for national legislation [50]. Recent Dutch initiatives like the "Electronic Data Exchange in Health Care Bill" [51] and national quality and information standards for the exchange of medication data [52,53] are important steps being taken for more integrated data at the point of care. However, the same level of attention remains needed to ensure that complete data is available for secondary uses. As the informants described, the actionability of primary care prescribing data sources demands looking beyond individual interventions. The possibility to link data from various sources and construct the whole patient journey from that data is needed.

Second, our findings suggest existing indicators require further development to avoid a one-size-fits-all approach. Indicators should be better differentiated by individual prescription and the information needs of stakeholders. This challenge is a characteristic of an indicator's fitness for purpose, that is, the precision with which an indicator aligns with the intended user's information needs [20]. Similar to previous studies (e.g. [13,54,55]), informants described differences in their desired type of information. These differences range from high-level volume indicators at the macro-level to detailed data, with patient-specific information on new and re-prescribing rates at the micro-level. We also found variability in information needs by prescription, for example, needing information about the appropriateness of antibiotics prescribed versus new, refills and long-term use of prescriptions in the case of benzodiazepines or opioids. The co-creation of indicators with healthcare providers, researchers, software vendors and the users of the data is needed and should be guided by the indicator's intended uses in terms of micro-meso-macro context and prescription type.

Third, the importance of linking up quality of care efforts from the national level to the local level is well-established [56-58]. In the absence of such links, policy priorities like managing antibiotic resistance and responding to the opioid epidemic, risk to remain solely high-level goals rather than cascading the system. However, available data and fit for purpose indicators alone will not guarantee that the information will be leveraged for learning and improvement at all levels. The analysis of

For peer review only - http://bmjopen.bmj.com/site/about/guidelines.xhtml

indicators, how that information is returned to end-users in reports or dashboards, and ultimately, processes for reflection on the information, need to be fostered and tailored to the different stakeholders.

We additionally note the following observations. First, despite the range of stakeholders and activities found at each level of the healthcare systems, descriptions around the use of prescribing data by the public and patients was absent. Relatedly, concerns around the public disclosure of information as a threat to an organization's performance was not a recurrent theme.

Strengths and limitations

This study was enriched by the diverse engagement of stakeholders across all levels of the Dutch healthcare system, resulting in a thorough qualitative dataset. The advanced digitalization and secondary uses of primary care data in the Dutch setting may be transferable to other data-rich contexts while also serving as an aspirational example for those at an earlier stage of development. For the purposes of this study and its scope, we focused on the use of indicators for antibiotics, benzodiazepines and opioids and the results, therefore, may not reflect the nuances of all prescription types. Other types of medications, such as for chronic conditions, were excluded as the management of healthcare needs is multifaceted and the appropriate rate of prescriptions is highly patient, disease and risk-factor specific. All interviews took place in English with native Dutch-speakers. Lastly, the study by design is exploratory in nature. Therefore, patterns and experiences by stakeholder and data types require testing with a larger sample, including patients, before they can be generalized.

Conclusions

Drawing on the expertise of the diverse sample of stakeholders interviewed, we described the information potential of electronic clinical, administrative, and claims prescribing data for secondary quality-related uses. Informants stressed the unique strengths and limitations of available data sources, with the incompleteness of each individually a key challenge. While primary care prescribing data is in use across the Dutch healthcare system, existing indicators require further development. In the case of antibiotics, this is found as a need to better indicate the appropriateness of prescriptions and for benzodiazepines and opioids, to monitoring their long-term use. Beyond methodological considerations about the indicators themselves, contextual considerations related to the information system and regulations as well as managerial considerations influencing an indicator's use in practice are areas identified for further prioritization. To curb societal concerns like antibiotic resistance and the misuse of opioids and benzodiazepines, the availability of prescribing data alone is insufficient. Available data sources must be linked and made actionable through fit for purpose and fit for use indicators applied at all levels of the healthcare system.

For peer review only - http://bmjopen.bmj.com/site/about/guidelines.xhtml

Contributions
EB, RV, LR, NK and DK conceptualised the study. EB with the support of RV and LR conducted data collection. EB prepared the manuscript. All authors provided feedback and contributed to revising the manuscript. All authors approved the final version.

Ethics and other permissions
The research adheres to the Dutch ethics guidelines stated in the 'Medical Research Act with People (Wet medisch-wetenschappelijk onderzoek met mensen (WMO)) (Dutch), in BWBR0009408, W.a.S. Ministry of Health, Editor. 1998: Hague, Netherlands',51) for which exception applies as no human data were retained and voluntary informed consent of participants was deemed adequate by the authors.

Funding
This work was carried out by the Marie Skłodowska-Curie Innovative Training Network for Healthcare Performance Intelligence Professionals (HealthPros) that has received funding from the European Union's Horizon 2020 research and innovation programme under grant agreement Nr. 765141.

Conflicts of interest
None declared.

Acknowledgements
We thank all participants who provided their time and expertise to this study as well as the time of the Data Expert Community members and reviews by HealthPros fellows, Karin Hek, and other Nivel colleagues.

Data sharing
The dataset supporting the conclusions of this study are included within the article and its supplementary files.

For peer review only

For peer review only - http://bmjopen.bmj.com/site/about/guidelines.xhtml

References

1. OECD. Stemming the Superbug Tide. Paris: OECD Publishing; 2018.
2. World Health Assembly. Global action plan on antimicrobial resistance. Geneva: World Health Organization; 2015.
3. United Nations Office on Drugs and Crime. World Drug Report 2019. Vienna, Austria: United Nations 2019. Contract No.: Booklet 1.
4. OECD. Addressing Problematic Opioid Use in OECD Countries. Paris: OECD Publishing; 2019.
5. Palinkas LA. Opioid Use Epidemic in Mexico: Global Solutions to a Global Problem. American journal of public health. 2019;109(1):26-7.
6. Hayhoe B, Lee-Davey J. Tackling benzodiazepine misuse. BMJ (Clinical research ed). 2018;362:k3208.
7. OECD. Health at a Glance 2019. Paris: OECD Publishing; 2019.
8. OECD. Safe prescribing in primary care. Health at a Glance 2021: OECD Indicators. Paris: OECD Publishing; 2021.
9. Bach P, Hartung D. Leveraging the role of community pharmacists in the prevention, surveillance, and treatment of opioid use disorders. Addiction Science & Clinical Practice. 2019;14(1):30.
10. Smith P, Mossialos E, Papanicolas I. Performance measurement for health system improvement: eperiences, challenges and prospects. Copenhagen: WHO Regional Office for Europe; 2008.
11. Mainz J. Defining and classifying clinical indicators for quality improvement. International Journal for Quality in Health Care. 2003;15(6):523-30.
12. Raleigh VS, Foot C. Getting the measure of quality: opportunities and challenges. London: The King's Fund; 2010.
13. Smith P, Mossialos E, Papanicolas I, Leatherman S. Part 1: Principles of performance measurement. In: Smith P, Mossialos E, Papanicolas I, Leatherman S, editors. Performance measurement for health system improvement: experiences, challenges and prospects. Copenahgen: WHO Regional Office for Europe; 2008.
14. van den Broek d'Obrenan J, Verheij TJM, Numans ME, van der Velden AW. Antibiotic use in Dutch primary care: relation between diagnosis, consultation and treatment. Journal of Antimicrobial Chemotherapy. 2014;69(6):1701-7.
15. Verheij RA, van der Zee J. Collecting information in general practice: 'just by pressing a single button'? Utrecht, the Netherlands: Nivel; 2006.
16. Kroneman M, Boerma W, van den Berg MJ, Groenewegen P, de Jong J, van Ginneken E. The Netherlands: health system review2016. 1–239 p.
17. OECD. Toward an integrated health information system in the Netherlands: Draft interim brief and recommendations. Paris: OECD Publishing; 2021.
18. Panch T, Pearson-Stuttard J, Greaves F, Atun R. Artificial intelligence: opportunities and risks for public health. The Lancet Digital Health. 2019;1(1):e13-e4.
19. Verheij RA, Curcin V, Delaney B, McGilchrist MM. Possible sources of bias in primary care electronic health record data use and reuse Journal of medical internet research. 2018;20(5).
20. Barbazza E, Klazinga N, Kringos D. Exploring the actionability of healthcare performance indicators for quality of care: a qualitative analysis of the literature, expert opinion and user experience BMJ Quality and Safety. 2021;30:1010-1020.
21. Menear M, Blanchette M-A, Demers-Payette O, Roy D. A framework for value-creating learning health systems. Health research policy and systems. 2019;17(1):79.
22. Friedman C, Rubin J, Brown J, Buntin M, Corn M, Etheredge L, et al. Toward a science of learning systems: a research agenda for the high-functioning Learning Health System. J Am Med Inform Assoc. 2015;22(1):43-50.
23. van der Bij S, de Hoon S, Nielen M, de Jong A, de Boer D, Verheij R. Routine recorded care data as a source of quality information [Dutch]. Utrecht, the Netherlands: Nivel; 2016.
24. Botje D, ten Asbroek G, Plochg T, Anema H, Kringos DS, Fischer C, et al. Are performance indicators used for hospital quality management: a qualitative interview study amongst health

For peer review only - http://bmjopen.bmj.com/site/about/guidelines.xhtml

professionals and quality managers in The Netherlands. BMC Health Services Research. 2016;16(1):574.
25. Giesen P, Willekens M, Mokkink H, Braspenning J, Van Den Bosch W, Grol R. Out-of-hours primary care: development of indicators for prescribing and referring. International journal for quality in health care : journal of the International Society for Quality in Health Care. 2007;19(5):289-95.
26. Bos V, Klazinga NS, Kringos DS. Improving performance intelligence for governing an integrated health and social care delivery network: a case study on the Amsterdam Noord district. BMC Health Serv Res. 2021;21(1):517.
27. RIVM. NethMap 2019: Consumption of antimicrobial agents and antimicrobial resistance among medically important bacteria in the Netherlands. RIVM; 2019.
28. Trimbos Institute. National Drug Monitor: Annual Report. Utrecht, the Netherlands: Trimbos Institute; 2019.
29. Rendle KA, Abramson CM, Garrett SB, Halley MC, Dohan D. Beyond exploratory: a tailored framework for designing and assessing qualitative health research. BMJ open. 2019;9(8):e030123-e.
30. Tong A, Sainsbury P, Craig J. Consolidated criteria for reporting qualitative research (COREQ): a 32-item checklist for interviews and focus groups. International Journal for Quality in Health Care. 2007;19(6):349-57.
31. Vogl S, Schmidt E, Zartler U. Triangulating perspectives: ontology and epistemology in the analysis of qualitative multiple perspective interviews. International Journal of Social Research Methodology. 2019;22(6):611-24.
32. Schweikardt C, Verheij RA, Donker GA, Coppieters Y. The historical development of the Dutch Sentinel General Practice Netowrk from a paper-based into a digital primary care monitoring system J Public Health. 2016.
33. Verheij R, Witvliet C, Jansen T, Hooiveld M, Hilten O van. Reuse of routine care data for policy and science: how things can be improved [Dutch]. Utrecht, the Netherlands: Nivel; 2019.
34. Braun V, Clarke V. Using thematic analysis in psychology. Qualitative Research in Psychology. 2006;3(2):77-101.
35. Meyer DZ, Avery LM. Excel as a Qualitative Data Analysis Tool. Field Methods. 2008;21(1):91-112.
36. Medical Research Act with People (Wet medisch-wetenschappelijk onderzoek met mensen (WMO)) [Dutch], (1998).
37. Stoker LJ, Heerdink ER, Janssen R, Egberts TCG. Effect of reimbursement restriction policy on the use of benzodiazepines in the Netherlands: an interrupted time series analysis. BMJ Open. 2019;9(9):e029148.
38. NIVEL. Nivel Primary Care Database: NIVEL 2019 [Available from: https://www.nivel.nl/en/nivel-primary-care-database.
39. Kollen BJ, van der Veen WJ, Groenhof F, Donker GA, van der Meer K. Discontinuation of reimbursement of benzodiazepines in the Netherlands: does it make a difference? BMC family practice. 2012;13(1):111.
40. Lareb NPC. About Lareb 2020 [Available from: https://www.lareb.nl/en/pages/about-lareb.
41. Verheij RA, Curcin V, Delaney BC, McGilchrist MM. Possible Sources of Bias in Primary Care Electronic Health Record Data Use and Reuse. J Med Internet Res. 2018;20(5):e185.
42. Ramerman L, Hek K, Sluiter R, Middelweerd A, Meijer A, Dijk L, et al. Formulary-oriented prescribing by the general practitioner: instrument development and monitoring 2018. Utrecht, the Netherlands: Nivel; 2020.
43. Trietsch J, van Steenkiste B, Grol R, Winkens B, Ulenkate H, Metsemakers J, et al. Effect of audit and feedback with peer review on general practitioners' prescribing and test ordering performance: a cluster-randomized controlled trial. BMC family practice. 2017;18(1):53.
44. Rowe TA, Linder JA. Novel approaches to decrease inappropriate ambulatory antibiotic use. Expert Rev Anti Infect Ther. 2019;17(7):511-21.
45. van Eeghen C, Kennedy AG, Pasanen ME, MacLean CD. A New Quality Improvement Toolkit to Improve Opioid Prescribing in Primary Care. The Journal of the American Board of Family Medicine. 2020;33(1):17.

For peer review only - http://bmjopen.bmj.com/site/about/guidelines.xhtml

46. Williams R, Ashcroft DM, Brown B, Rojas E, Peek N, Johnson O. Process Mining in Primary Care: Avoiding Adverse Events Due to Hazardous Prescribing. Studies in health technology and informatics. 2019;264:447-51.
47. Okoli GN, Myles P, Murray-Thomas T, Shepherd H, Wong ICK, Edwards D. Use of Primary Care Data in Research and Pharmacovigilance: Eight Scenarios Where Prescription Data are Absent. Drug Safety. 2021.
48. Saigí-Rubió F, Pereyra-Rodríguez JJ, Torrent-Sellens J, Eguia H, Azzopardi-Muscat N, Novillo-Ortiz D. Routine Health Information Systems in the European Context: A Systematic Review of Systematic Reviews. International Journal of Environmental Research and Public Health. 2021;18(9).
49. Bogaert P, Verschuuren M, Van Oyen H, van Oers H. Identifying common enablers and barriers in European health information systems. Health Policy. 2021;125(12), 1517-1526.
50. Hansen J, Wilson P, Verhoeven E, Kroneman M, Kirwan M, Verheij R, et al. Assessment of the EU Member States' rules on health data in light of GDPR. Brussels: European Commission; 2021.
51. Rules regarding the electronic sharing and access of data between healthcare providers in designated data exchanges (Electronic Data Exchange in Healthcare Act), (2021).
52. Directive Transfer of Medication data in the chain Review 2018/2019, (2019).
53. Nictiz. Information standard: digital recipe traffic NEN7503 2021 [Available from: https://www.nictiz.nl/standaardisatie/informatiestandaarden/medicatieveiligheid/Informatiestandaard/#functioneel.
54. Damberg CL, Sorbero ME, Lovejoy SL, Lauderdale K, Wertheimer S, Smith A, et al. An Evaluation of the Use of Performance Measures in Health Care. Rand health quarterly. 2012;1(4):3-.
55. Palin V, Tempest E, Mistry C, van Staa TP. Developing the infrastructure to support the optimisation of antibiotic prescribing using the learning healthcare system to improve healthcare services in the provision of primary care in England. BMJ Health Care Inform. 2020;27(1).
56. Jeremy Veillard THSASKNSK, Adalsteinn DB. Making Health System Performance Measurement Useful to Policy Makers: Aligning Strategies, Measurement and Local Health System Accountability in Ontario. Healthcare Policy. 2010;5(3):49-65.
57. Travis P, Egger D, Davies P, Mechbal A. Towards better stewardship: concepts and critical issues. Geneva: World Health Organization; 2002.
58. Baez-Camargo C. Accountability for better healthcare provision: A framework and guidelines to define, understand and assess accountability in health systems. Basel: Institute on Governance; 2011.

For peer review only - http://bmjopen.bmj.com/site/about/guidelines.xhtml

Optimizing the secondary use of primary care prescribing data to improve quality of care: a qualitative analysis

Supplementary file 1: Interview topic guide

1. Purpose of use of primary care prescribing data

- How does your organization currently use primary care prescribing data? Refer to the table on the following page (Supplementary file 2) listing core actors and purposes of use identified. Is this accurate and complete?
- How would you describe the information you need to carry-out your organization's role? (e.g. multi-year information on performance at national-level; aggregate, comparative performance measures on providers; timely, continuous information at patient-level, etc.).
- What actors do you work with directly in the scope of primary care prescribing data?

2. Current use of prescribing data

- **Use of indicators**. Does your organization actively collect data related to the following: (1) Antibiotics; (2) Opioids; (3) Benzodiazepines. If so, what are the indicators or measures used related to each? How long have these been reported on? Is it intended for internal or external use? Who is the target audience (intended user) of the information generated?
- **Data sources**. What is your primary source of primary care prescribing data? (e.g. medical records, administrative data, specific research database, others). How is the data collected? If other actors are involved, whom does this include? Is the data considered of quality?
- **Analysis.** How is the data currently analyzed – benchmarking, time trend, international comparison? How would you describe this analysis? (e.g. time interval, comparators used, aggregation as composite scores, etc.)
- **Dissemination**. How is the data disseminated? What is the format of reporting (print, electronic, web-based)? What is the lag time in presenting analyzed data? How does it reach the intended target audience?

3. Perceived actionability

- In your opinion, how can the process in which data is analyzed and reported on be improved upon?
- Is the information generated useful for your purposes? That is, are you able to make decisions and learn from the information?
- In general, what are the obstacles to the optimal use of primary care prescribing data at present?

For peer review only - http://bmjopen.bmj.com/site/about/guidelines.xhtml

Supplementary file 2: Mapping of stakeholders and uses of prescribing data

Purpose of use[1]	Stakeholders
**Micro-level**	
Individual professional performance	Individual GP and HIS supplier<br>Community pharmacist and HIS supplier
Practice improvement	GP practice/peers using HIS<br>GP practice and insurers<br>GP practice and affiliate research networks<br>Community pharmacy and pharmacy network
Multidisciplinary improvement	Pharmacotherapy audit groups (GPs and pharmacists) (FTOs)
**Meso-level**	
Organization/ networks performance improvement	Care groups and affiliate GP practices (e.g., MCC Omens Care Group, Zorg In Ontwikkeling)
Quality-based financing	Health Insurers (e.g., Zilveren Kruis)
Monitoring	Lareb Side Effects Center<br>Foundation of Pharmaceutical Statistics (SFK)
Professional development	Dutch Institute for Responsible Drug Use (IVM)<br>National Association of GPs (LHV)
Advocacy and standards	Dutch GP Association (NHG)<br>The Royal Dutch Society for the Promotion of Pharmacy (KNMP)<br>Organization for first line care (InEen)<br>Patient Federation Netherlands (Patienten Federatie)
**Macro-level**	
Strategy development	Ministry of Health, Welfare and Sport
System performance	National Institute for Public Health and the Environment (RIVM)
System quality assurance	Medicines Evaluation Board (MEB)<br>National Health Care Institute (ZiNL)<br>Health Care Inspectorate (IGJ)<br>Dutch Healthcare Authority (NZa)
**Cross-cutting**	
	Netherlands Institute for Health Services Research (Nivel)<br>Institute for Drug Outcomes Research (Pharmo)<br>Vektis<br>Nictiz<br>Digitalis

[1]Purposes of use draw from the study findings: Barbazza E, Klazinga NS, Kringos DS. Exploring the actionability of healthcare performance indicators for quality of care: a qualitative analysis of the literature, expert opinion and user experience. BMJ Quality & Safety 2021;30:1010-1020.

For peer review only

For peer review only - http://bmjopen.bmj.com/site/about/guidelines.xhtml

Supplementary file 3: Characteristics of informants

#	Code	Level	Stakeholder	Gender	Format
1	Association–1	Meso	The Royal Dutch Society for the Promotion of Pharmacy (KNMP)	Female	Phone
2	Health professional–2	Micro	Health Professional	Male	Phone
3	Care group–3	Meso	MCC Omens Care Group	Female	Phone
4	Care group–4	Meso	ZIO	Female	Phone
5	Government–5	Macro	Medicines Evaluation Board (MEB)	Male	Phone
6	EHR supplier–6	Cross-cutting	Digitalis	Male	In-person
7	Association–7	Meso	Organization of Firstline Care (InEen)	Male	Phone
8	Association–8	Meso	Dutch Institute for Responsible Drug Use (IVM)	Female	Phone
9	EHR supplier–9	Cross-cutting	Nictiz	Male	Written
10	EHR supplier–10	Cross-cutting	CampuGroup Medical (CGM)	Male	Phone
11	Government–11	Macro	Ministry of Health, Welfare and Sport	Male	Phone
12	Association–12	Meso	Lareb Side Effects Center	Male	Phone
13	Association–13	Meso	Foundation for Quality Indicators Pharmacy (SFK)	Male	Phone
14	Government–14	Macro	National Institute for Public Health and the Environment (RIVM)	Male	Written
15	Association–15	Meso	Dutch General Practitioners Association (NHG)	Male	Phone
16	Government–16	Macro	Health Care Inspectorate	Male	Phone
17	Research–17	Cross-cutting	Institute for Drug Outcomes Research (Pharmo)	Male	Phone
18	Association–18	Meso	Patient Federation	Male	Phone
19	Insurer–19	Meso	Zilveren Kruis	Male	Phone
20	Association–20	Meso	National General Practitioners Association (LHV)	Male	Phone
21	EHR supplier–21	Cross-cutting	Vektis	Male	Phone
22	Government agency–22	Macro	National Health Care Institute	Male	Phone
23	Government agency–23	Macro	Ministry of Health, Welfare and Sport	Male	Phone
24	Government agency–24	Macro	Dutch Healthcare Authority	Female	Phone
25	Government agency–24	Macro	Dutch Healthcare Authority	Male	Phone
26	Research–25	Cross-cutting	Netherlands Institute for Health Services Research	Female	In-person
27	Research–25	Cross-cutting	Netherlands Institute for Health Services Research	Female	In-person
28	Government agency–26	Macro	National Institute for Public Health and the Environment (RIVM)	Female	Phone

EHR: Electronic health record.

For peer review only

For peer review only - http://bmjopen.bmj.com/site/about/guidelines.xhtml

Optimizing the secondary use of primary care prescribing data to improve quality of care: a qualitative analysis

Consolidated Criteria for Reporting Qualitative Research (COREQ) Checklist[a]

#	Item	Question/description	Answer	Section (page, para)
**Domain 1: Research team and reflexivity**				
**Personal Characteristics**				
1	Interviewer/ facilitator	Which author/s conducted the interview or focus group?	EB	Methods (pg 5, para 1)
2	Credentials	What were the researcher's credentials?	BHSc, MSc, PhD candidate	Methods (pg 5, para 1)
3	Occupation	What was their occupation at the time of the study?	HealthPros Fellow and PhD candidate<br>Department of Public and Occupational Health<br>University of Amsterdam	Methods (pg 5, para 1)
4	Gender	Was the researcher male or female?	Female	Title page
5	Experience and training	What experience or training did the researcher have?	At the time of the interviews, had completed more than five years of interview-based data collection.	Methods (pg 5, para 1)
**Relationship with participants**				
6	Relationship established	Was a relationship established prior to study commencement?	Yes, informants were contacted via email and corresponded with the interviewer to confirm their interest and agreement to participate.	Methods (pg 5, para 4)
7	Participant knowledge of the interviewer	What did the participants know about the researcher? e.g. personal goals, reasons for doing the research.	Participants received a study brief prior to the interview detailing the study's purpose, aims, funding and dissemination of results as well as the questions to be discussed.	Methods (pg 5, para 5), Supplementary file 1
8	Interviewer characteristics	What characteristics were reported about the interviewer/facilitator? e.g. Bias, assumptions, reasons and interests in the research topic	EB acknowledged her role as a research fellow on health care performance intelligence and that the study was being conducted in the scope of her PhD research.	Methods (pg 5, para 5)
**Domain 2: study design**				
**Theoretical framework**				
9	Methodological orientation and Theory	What methodological orientation was stated to underpin the study? e.g. grounded theory, discourse analysis, ethnography,	Exploratory study design using multiple perspective semi-structured interviews and thematic analysis.	Methods (pg 5, para 1)

For peer review only

#	Item	Question/description	Answer	Section (page, para)
		phenomenology, content analysis		
**Participant selection**				
10	Sampling	How were participants selected? e.g. purposive, convenience, consecutive, snowball	Purposive including snowballing and then sampling to sufficiency	Methods (sample and recruitment selection, pg 5 para 3)
11	Method of approach	How were participants approached? e.g. face-to-face, telephone, mail, email	Via email	Methods (pg 5, para 4)
12	Sample size	How many participants were in the study?	28 informants	Results (pg 7, para 1)
13	Non-participation	How many people refused to participate or dropped out? Reasons?	Non-participants (n=25) either were unreachable (n=12), referred to an alternative contact (n=10) that was met with to follow or were unavailable due to time constraints (n=3).	Results (pg 7, para 1)
**Setting**				
14	Setting of data collection	Where was the data collected? e.g. home, clinic, workplace	Interviews took place by telephone and in-person.	Methods (pg 5, para 5)
15	Presence of non-participants	Was anyone else present besides the participants and researchers	No non-participants were present during data collection.	Results (pg 7, para 1)
16	Description of sample	What are the important characteristics of the sample? e.g. demographic data, date	Table 1 profiles the panelists by their uses of primary care prescribing data, affiliate organizations and gender	Results (pg 7, para 1)
**Data collection**				
17	Interview guide	Were questions, prompts, and guides provided by the authors? Was it pilot tested?	Participants received in advance of the interview the key questions in written form.	Methods (pg 5, para 5)
18	Repeat interviews	Were repeat interviews carried out? If yes, how many	No	Results (pg 7, para 1)
19	Audio/visual recording	Did the research use audio or visual recording to collect the data?	Yes, interviews were audio recorded in agreement with informants.	Methods (pg 5, para 5)
20	Field notes	Were field notes made during and/or after the interview or focus group?	No	Methods (pg 5, para 5)
21	Duration	What was the duration of the interviews or focus groups?	Interviews lasted between 30–60 minutes	Methods (pg 5, para 5)
22	Data saturation	Was data saturation discussed?	Yes, data saturation was reached when stakeholders spanning the differentiated uses of primary care prescribing data were met.	Methods (pg 5, para 5)
23	Transcripts returned	Were transcripts returned to participants for comment and/or correction?	No.	Methods (pg 6)
**Domain 3: analysis and findings**				

For peer review only

#	Item	Question/description	Answer	Section (page, para)
**Data analysis**				
24	Number of data coders	How many data coders coded the data?	EB	Methods (pg 5-6)
25	Description of the coding tree	Did authors provide a description of the coding tree?	Yes, the main level codes are reported.	Methods, data analysis (pg 5-6)
26	Derivation of themes	Were themes identified in advance or derived from the data?	Main level codes were derived from an existing classification of actionable healthcare performance indicators. Unrestricted coding was applied to identify new themes.	Methods, data analysis (pg 5-6)
27	Software	What software, if applicable, was used to manage the data?	Microsoft Word and Excel	Methods, data analysis (pg 5-6)
28	Participant checking	Did participants provide feedback on the findings	Results were shared publicly at a conference.	Methods (pg 6)
**Reporting**				
29	Quotations pr-esented	Were participant quotations presented to illustrate the themes/findings? Was each quotation identified? e.g. participant number	Yes, throughout results. Confidentiality was assured by referring to informants by stakeholder type and a randomly assigned number (e.g., Health professional–1).	Results (pg 7-13)
30	Data and findings consistent	Was there consistency between the data presented and the findings?	Findings are presented in the approach of the research questions and interview topic guide.	Results (pg 7-13)
31	Clarity of major themes	Were major themes clearly presented in the findings?	Tables are used to present major themes. Subheadings are used to improve clarity.	Results (pg 7-13)
32	Clarity of minor themes	Is there a description of diverse cases or discussion of minor themes?	Findings are supplemented in-text by quotes and descriptions of sub-themes.	Results (pg 7-13)

[a] Tong A, Sainsbury P, Craig J. Consolidated criteria for reporting qualitative research (COREQ): a 32-item checklist for interviews and focus groups. Int J Qual Health Care 2007;19:349–57.

For peer review only

BMJ Open

Optimising the secondary use of primary care prescribing data to improve quality of care: a qualitative analysis

Journal:	*BMJ Open*
Manuscript ID	bmjopen-2022-062349.R1
Article Type:	Original research
Date Submitted by the Author:	14-Jun-2022
Complete List of Authors:	Barbazza, Erica; Amsterdam UMC Locatie AMC, Department of Public and Occupational Health<br>Verheij, Robert A; Netherlands Institute for Health Services Research, Learning Health Systems Research Programme<br>Ramerman, Lotte; Netherlands Institute for Health Services Research, Learning Health Systems Research Programme<br>Klazinga, Niek; Amsterdam UMC Locatie AMC, Department of Public and Occupational Health<br>Kringos, D; Amsterdam UMC Location AMC, Department of Public and Occupational Health
**Primary Subject Heading**:	Health services research
Secondary Subject Heading:	Health services research, Qualitative research, General practice / Family practice
Keywords:	PRIMARY CARE, Quality in health care < HEALTH SERVICES ADMINISTRATION & MANAGEMENT, QUALITATIVE RESEARCH, Health & safety < HEALTH SERVICES ADMINISTRATION & MANAGEMENT, Information management < BIOTECHNOLOGY & BIOINFORMATICS

SCHOLARONE™
Manuscripts

I, the Submitting Author has the right to grant and does grant on behalf of all authors of the Work (as defined in the below author licence), an exclusive licence and/or a non-exclusive licence for contributions from authors who are: i) UK Crown employees; ii) where BMJ has agreed a CC-BY licence shall apply, and/or iii) in accordance with the terms applicable for US Federal Government officers or employees acting as part of their official duties; on a worldwide, perpetual, irrevocable, royalty-free basis to BMJ Publishing Group Ltd ("BMJ") its licensees and where the relevant Journal is co-owned by BMJ to the co-owners of the Journal, to publish the Work in this journal and any other BMJ products and to exploit all rights, as set out in our licence.

The Submitting Author accepts and understands that any supply made under these terms is made by BMJ to the Submitting Author unless you are acting as an employee on behalf of your employer or a postgraduate student of an affiliated institution which is paying any applicable article publishing charge ("APC") for Open Access articles. Where the Submitting Author wishes to make the Work available on an Open Access basis (and intends to pay the relevant APC), the terms of reuse of such Open Access shall be governed by a Creative Commons licence – details of these licences and which Creative Commons licence will apply to this Work are set out in our licence referred to above.

Other than as permitted in any relevant BMJ Author's Self Archiving Policies, I confirm this Work has not been accepted for publication elsewhere, is not being considered for publication elsewhere and does not duplicate material already published. I confirm all authors consent to publication of this Work and authorise the granting of this licence.

For peer review only

Original research

Optimising the secondary use of primary care prescribing data to improve quality of care: a qualitative analysis

Authors
Erica Barbazza MSc[1], https://orcid.org/0000-0001-7621-1638
Robert Verheij MSc PhD[2], https://orcid.org/0000-0003-2234-4819
Lotte Ramerman MSc PhD[2], https://orcid.org/0000-0001-8436-0924
Niek Klazinga MD PhD[1], https://orcid.org/0000-0002-3937-8014
Dionne Kringos MSc PhD[1], https://orcid.org/0000-0003-2711-4713

[1]Department of Public and Occupational Health, Amsterdam UMC, University of Amsterdam, Amsterdam Public Health research institute, Amsterdam, the Netherlands
[2]Learning Health Systems Research Programme, Netherlands Institute for Health Services Research, Utrecht, the Netherlands

Address for correspondence
Erica Barbazza
Department of Public and Occupational Health
Amsterdam UMC, Academic Medical Centre
University of Amsterdam
Meibergdreef 9, 1105 AZ, Amsterdam, the Netherlands
Email: e.s.barbazza@amsterdamumc.nl
Tel.: +45 31 12 17 11

Word count abstract: 298 words

Word count main text: 4,841 (excluding figures, tables, references)

Figures and tables: 3

Supplementary material
COREQ checklist
Supplementary file 1: Interview topic guide
Supplementary file 2: Mapping of stakeholders and uses of prescribing data
Supplementary file 3: Characteristics of informants

Running title
Secondary uses of primary care prescribing data

Keywords
primary care; prescriptions; quality indicators; performance measures; the Netherlands; health information management

For peer review only - http://bmjopen.bmj.com/site/about/guidelines.xhtml

Abstract

Objectives. To explore available data sources, secondary uses, and key considerations for optimising the actionability of primary care prescribing data to improve quality of care in the Dutch context.

Design. An exploratory qualitative study was undertaken based on semi-structured interviews. We anchored our investigation around three tracer prescription types: antibiotics; benzodiazepines; and opioids. Descriptive and explanatory themes were derived from interview data using thematic analysis.

Setting. Stakeholders were sampled from across the micro (clinical), meso (organisational), and macro (policy) contexts of the Dutch primary care system.

Participants. The study involved 28 informants representing general practitioners (GPs), community pharmacists, regional chronic care networks (care groups), academia and research institutes, insurers, professional associations, electronic health record (EHR) vendors, and national authorities.

Results. In the Netherlands, three main sources of data for improving prescribing in primary care are in use: clinical data in the EHRs of GP practices; pharmacy data in community pharmacy databases; and claims data of insurers. While the secondary use of pharmacy and claims data is well-established across levels, the use of these data together with EHR data is limited. Important differences in the types of prescribing information needed by micro-meso-macro context are found, though the extent to which current indicators address these varies by prescription type. Five main themes were identified as areas for optimising data use: (1) measuring what matters, (2) increasing data linkages, (3) improving data quality, (4) facilitating data sharing, and (5) optimising fit for use analysis.

Conclusions. To make primary care prescribing data useful for improving quality, consolidated patient-specific data on the indication for a prescription and dispensed medicine, over time, is needed. In the Netherlands, the selection of indicators requires further prioritisation to better signal the appropriateness and long-term use of prescription drugs. Prioritising data linkages is critical towards more actionable use.

For peer review only - http://bmjopen.bmj.com/site/about/guidelines.xhtml

Strengths and limitations of this study

- Semi-structured interviews elicited first-hand insights into the secondary use of primary care prescribing data, filling this knowledge gap in the published literature.
- Stakeholder interviews spanned all levels of the Dutch healthcare system and engaged varied perspectives, including community pharmacists and general practice, offering diverse insights.
- Three tracer prescription types were selected to anchor discussions with stakeholders and the findings may not capture the nuances of all prescriptions.
- Our study is deliberatively exploratory in nature, thus patterns and experiences by stakeholder types require testing with a larger sample, including patients, before they can be generalised.

For peer review only

For peer review only - http://bmjopen.bmj.com/site/about/guidelines.xhtml

Introduction

Improving prescribing practices has received increasing policy attention globally. This prioritisation follows concerning trends, including rising levels of antimicrobial resistance [1,2], an epidemic of opioid use [3-5], and the increasing misuse of benzodiazepines [6-8]. In the Dutch context—like other gatekeeping models of primary care—general practitioners (GPs) function as the first-line for patient management and entry-point to secondary healthcare services. In effect, GPs together with community-based pharmacists are central to services including the issuing and refilling of outpatient prescription medicines [9]. Measuring the performance of services provided by GPs and community pharmacists (both key primary care providers) is fundamental to improve quality [10]. Hence, the use of quality indicators, as a measurement tool to quantify quality, is of critical importance [11-13].

In the Netherlands, the far-reaching digitalisation of patient data and physician prescribing has long been recognised as a powerful resource for improving quality [14-16]. All GP practices (approximately 5,000) record data in electronic health records (EHRs) supplied by ten main EHR vendor brands on the market [16]. Since 2014, primary care prescriptions are issued electronically for dispensing medicines at one of approximately 2,000 community pharmacies across the country [16]. The resulting electronic primary care prescribing data has secondary uses that extend across the micro (clinical care), meso (organisations and networks), and macro (policy) context of the Dutch healthcare system [17].

However, as health services research has increasingly called attention to, the availability of data alone does not guarantee its *use* for quality of care-related decision-making [18,19]. The information produced should also be actionable [20]. The movement towards learning healthcare systems further attests to the critical role of actionable data as an integral part of healthcare delivery processes [21,22]. In primary care, given the critical potential of prescription data to indicate, for instance, inappropriate prescriptions, overprescribing, addiction issues, or antimicrobial resistance trends, it is essential to ensure healthcare systems are optimally using available prescription data for learning and decision-making purposes towards quality improvement in practice.

In the data-rich context of the Netherlands, activity around the use of healthcare data is high: survey data finds Dutch GPs regularly receive as many as ten different feedback reports [23]. This volume of activity has called into question the extent to which performance indicators are actually used for improvement purposes. Research on the secondary uses of healthcare data has been conducted in the context of Dutch hospitals [24], out-of-hours care [25], and integrated care networks [26]. In the absence of an overview of routine primary care prescribing data sources, what and how available data is used for learning and improvement purposes across the healthcare system is unclear.

In this study, we set out to investigate the current secondary uses of primary care prescribing data for improving quality of care through the first-hand insights of stakeholders across the Dutch healthcare system. We also aimed to distil their views on opportunities to improve the use of prescribing data for quality of care-related decision-making. Importantly, the optimisation of secondary uses of primary care prescribing data is an intermediary step to improving care. Direct uses of prescribing data for patient care, such as for education purposes and shared decision-making, is also a key aspect to improve prescribing [27-29], however, these uses are outside the scope of this study. To anchor our investigation and generate concrete, practical examples of prescribing data uses, we focused on three commonly prescribed types of prescriptions: antibiotics; benzodiazepines; and opioids. The prescriptions are each of significant societal and public health importance [30,31] and vary in their etiological and therapeutic use (infection control, psychological disorders, and pain management, respectively). In combination, the selected prescription types can offer insights into the use of primary care prescribing data as a whole.

With this aim and focus, the study is guided by the following three questions: what are the available sources and characteristics of primary care prescribing data? How is this data currently used for improving quality of care? And, what are key considerations for optimising the secondary uses of primary care prescribing data?

For peer review only - http://bmjopen.bmj.com/site/about/guidelines.xhtml

Methods

Design

An exploratory qualitative study design was employed [32]. Reporting adheres to the Consolidated Criteria for Reporting Qualitative Research [33]. Semi-structured interviews with stakeholders ranging the clinical (micro), organisational (meso) and policy (macro) context of the Dutch healthcare system were conducted for rich individual exchanges and practical insights across the healthcare system [34]. The research team included experts on healthcare performance intelligence, primary care, health information systems, and the Dutch context. The primary researcher and interviewer is an experienced qualitative researcher and doctoral student on the actionability of healthcare performance indicators.

To operationalise the construct of actionable indicators, we drew from an existing definition depicting actionability as the two related constructs of *fitness for purpose*—information serving an intended decision-making function—and *fitness for use*—the ability to get the right information, into the right hands at the right time [20]. To explore fitness for purpose, the definition's differentiation of types of uses of indicators across healthcare systems was applied. This depiction of actionable indicators, together with our three main research questions, served as the framework for our interview guide. Specifically, the themes explored with informants included: sources of primary care prescribing data; current uses of prescribing data (anchored in the selected prescription types); and perceived actionability constraints (Supplementary file 1).

Sample and recruitment selection

We defined our target informants by Dutch stakeholders across the micro-meso-macro contexts of the healthcare system with first-hand use of primary care prescribing data for monitoring, assessing and/or improving quality. We identified more than 20 different stakeholders, ranging: government health agencies; associations, including patient and professional groups; regional care networks; health professionals; EHR suppliers; insurers; and researchers (Supplementary file 2). An initial listing was prepared based on reviews of key literature [16,35,36] and the expertise of the study team. The list was validated with an existing Dutch network (Data Expert Community), with representation of national stakeholders working in the field of healthcare data. Feedback from the network was solicited at an in-person meeting in November 2019 in Utrecht, the Netherlands.

We used multiple methods to reach prospective informants affiliated to the stakeholders identified. First, we reviewed the webpages of target stakeholders for contacts and membership lists. Second, the authorship of literature related to primary care and medicines in the Dutch context (eg, scientific articles, reports, evaluations, factsheets, presentations) was extracted. Third, the expertise of the study team and advice of external experts was solicited, and a snowballing approach was applied. In a similar way, some prospective participants served as contact mediating informants, suggesting alternative colleagues best suited for participating. Informants were invited to participate in the study via email by the authors (EB,RV,LR) and received a document detailing the background, aim, scope, and research questions.

Data collection

Interviews were conducted over a four-month period (November 2019 to February 2020). Interviews ranged 30–60 minutes in length. They were conducted both in-person and at-distance by phone, based on the proximity and preference of informants. In instances where informants requested to extend an invitation to colleagues, these interviews were conducted jointly. We also accommodated requests to answer questions in writing. With the agreement of informants, interviews were recorded and transcribed verbatim. Regular meetings with the full study team were organised to discuss the process and recurrent themes. The interviews were considered complete when the range of informants represented stakeholders spanning the micro-meso-macro levels of the healthcare system.

Data analysis

Thematic analysis was used to analyse interview data [37] in an Excel tool developed in the approach of Meyer and Avery [38]. The analysis process included familiarisation with the data, development of a coding framework, coding, mapping and interpretation of results. The coding framework was

For peer review only - http://bmjopen.bmj.com/site/about/guidelines.xhtml

developed based on the items of the semi-structured interviews: purposes of use; actors; indicators; data sources; analysis; dissemination; barriers; and opportunities for improvement (Supplementary file 2). Additional themes were generated through open (unrestricted) coding in an inductive approach. The initial coding and clustering of themes was conducted by the primary researcher. To ensure validity of the findings, the results were regularly reviewed by the full study team. In reporting on the results by research question, verbatim quotes were extracted from the transcripts.

Ethics

The research protocol was developed in accordance with the ethical requirements of the primary research affiliation to Amsterdam University Medical Centers of the University of Amsterdam and relevant Dutch ethics guidelines [39]. To ensure informed voluntary participation, informants contributing to this study provided written informed consent to participate during the recruitment stage and restated their consent verbally at the start of interviews. All interview data has been anonymized. Confidentiality was assured by referring to informants by stakeholder type and an assigned number (e.g., Health professional–1).

Patient and public involvement

The preliminary findings were shared at an international scientific conference in 2021. The interaction with participants provided a unique opportunity for critically reflecting on the findings.

For peer review only

For peer review only - http://bmjopen.bmj.com/site/about/guidelines.xhtml

Results

Characteristics of informants

In total, 53 informants were contacted of which 28 were interviewed representing 26 different stakeholders. Ten prospective informants referred to an alternative contact within their team or organisation. Non-participants were either unresponsive (n=12) or unavailable due to time constraints (n=3). In either instance (contact mediating informants or non-participants), no healthcare system level or type of stakeholder was overly non-responsive to participation. See Supplementary file 3 for a detailed breakdown.

Two interviews were conducted with two informants present. In two other instances, information was collected via email exchange only, at the preference of the informant. No repeat interviews were carried out. Some informants held multiple affiliations. Notably, three informants were both health professionals and affiliated to another stakeholder, as signalled by totals included in round brackets in Table 1. For the purposes of reporting, only one primary affiliation has been used (Table 1). See Supplementary file 3 also for a detailed overview of informant characteristics.

Table 1. Summary of informant characteristics

Characteristics	Total informants N=28	
	n[a]	%
**Healthcare system level (context)**		
Micro (clinical)	1 (4)	4
Meso (organisational)	11	39
Macro (policy)	9	32
Cross-cutting (research, EHR supplier)	7	25
**Type of stakeholder**		
Association (patient, professional)	8	29
Care group (network)	2	7
Government health agency	9	32
Health professional	1(4)	4
EHR supplier	4	14
Insurer	1	4
Research	3	11
**Gender**		
Female	8	29
Male	20	71

EHR: Electronic health record.
[a]Numbers in round brackets indicate the total number of informants when individuals with multiple affiliations are accounted for.

Sources and characteristics of primary care prescribing data

Three main sources of primary care prescribing data for secondary uses towards improving quality are in use in the Netherlands: clinical data in the EHRs of GP practices and dispensing data related to prescriptions dispensed in community pharmacy databases and claims for prescriptions of insurers.

Datasets which can be combined and supplemented with other information are available, specifically: the Institute for Drug Outcomes Research Database [40], Nivel Primary Care Database [15,35,41], and various research-specific datasets of academic networks of GPs (eg, Registration Network Groningen [42]). These datasets have the advantage of more complete information (diagnosis and dispensed medicines) though are limited to the voluntary participation GP practices. Other types of prescribing data though not specific to primary care include self-reported or physician-reported medicines' side effects [43] and in-patient prescribing in hospital databases.

For peer review only - http://bmjopen.bmj.com/site/about/guidelines.xhtml

Table 2 summarises these data sources, the nature of information and advantages, and limitations of each for secondary quality-related uses as described by informants. According to informants, not one data source is considered *complete*, as each has unique advantages, but also limitations as a potential source for quality-related decision-making. For example, clinical data in EHRs captures the diagnosis (indication) for a prescription, however, depending on the EHR system, it can lack details on the medicines retrieved and dispensed in community pharmacies. Conversely, administrative pharmacy data and insurance claims are rich in details of prescriptions dispensed and reimbursed, though lack clinical details found in EHRs, specifically associated laboratory results and a specific diagnosis. As informants described:

> The missed link between the diagnosis in the EHR and what is dispensed as the medication, leaves little insights into whether the prescription provided was the right one or necessary. (Health professional–2)

> From the pharmacist perspective, the absence of a link to a specific diagnosis means that interpreting values requires in most instances more analysis and reflection. (Association–13)

For peer review only

For peer review only - http://bmjopen.bmj.com/site/about/guidelines.xhtml

Table 2. Primary care prescribing data landscape in the Netherlands according to informants

Data source	Repository	Coverage	Nature of information	Advantages	Limitations
Clinical	EHRs	All GP practices	Prescription level data with patient ids including complete medical history, diagnosis, lab tests and prescribed medicines.	Includes indication for prescription. Possibility to link across databases using unique patient identifier. Possible to link with comorbidities.	Lacks data on prescriptions filled and dispensed by pharmacist. No central database. Varied recording of data across EHR suppliers.
Pharmacy dispensing data of community pharmacist	Foundation of Pharmaceutical Statistics	Across community pharmacies	Patient-level information on dispensed medicines in pharmacy system, medication including type, dosage, other medications.	Complete overview of dispensed medicines by community pharmacies.	Lacks data on diagnosis and lab results. Excludes: prescriptions issued but not retrieved; over-the-counter medicines; prescriptions issued and dispensed in hospitals.
Claims (pharmacy, services)	Drug Information Project (Dutch Health Care Institute)	Across community pharmacies	Information on prescription (e.g., dosage, quantity dispensed), prescriber, dispensing pharmacy and price declared/ reimbursed filled by public pharmacies.	Data collected across all practices/public pharmacies.	Lacks data on diagnosis. Includes data only for reimbursed medicines and services.
Other reposit-ories	Nivel Primary Care Database (Nivel)	Affiliate GP practices from across the country[1]	Data on consultations, diagnosis, prescribed medicines, with the possibility to link other data sources for environmental characteristics, migration background, income, insurance claims, pharmacy data.	Possibility to combine and supplement EHR data with information about pharmaceutical care and secondary level care.	EHR data from affiliated practices only, though representation across the country (10% of the population).
	Pharmo Data Network (Pharmo)	Affiliate care groups[2]	Linked data from public pharmacy database, GP database, hospital pharmacy databases, clinical laboratories.	Possibility to link to EHR data to administrative insurance claims data and pharmacy data.	Data from affiliate care groups only.
	Academic GP network databases	Networks in catchment area of large university hospitals	Patient-level data including complete medical history, diagnosis, medications, etc. for affiliated practices.	Includes indication for prescription. Possibility to link across databases using unique patient identifier.	Limited to affiliate GP practices. Research-specific uses of data.
	Vektis database (Vektis)	Across health care insurers	Insurers claims database of all reimbursed services with data on physician services (e.g., reason for visit) and procedures (e.g., tests).	Completeness of database, with data spanning across the Dutch population and insurers.	Lacks data on diagnosis. Includes data only for reimbursed medicines and services.

Notes: EHR=electronic health record; GP=general practitioner; Nivel=Netherlands Institute for Health Services Research; Pharmo= Institute for Drug Outcomes Research Database. [1]Approximately 500 GP practices, 1.7 million patients; [2]Approximately 13 care groups, 4 million patients.

For peer review only - http://bmjopen.bmj.com/site/about/guidelines.xhtml

Secondary uses of primary care prescribing data

The secondary uses and sources of primary care prescribing data are summarised to follow. See Supplementary file 1 for a detailed table. These descriptions are anchored in the illustrative prescription types applied. At the outset, the information needs by decision-making context and prescription type were described by informants (Table 3).

Table 3. Examples of information needs by type of prescription as described by informants

Context	Antibiotics	Benzodiazepines	Opioids
**Macro** (policy)	What is the overall volume of antibiotics prescribed annually?	How many elderly patients have a long-term benzodiazepine prescription?	What is the overall volume of opioids prescribed? How many are chronic opioid users?
**Meso** (organisa-tional)	How does the volume of prescribing compare with previous years? (care groups)	How does the volume of prescribing compare with previous years and age groups?	How does the volume of prescribing compare with previous years and age groups?
**Micro** (clinical)	Have I prescribed antibiotics appropriately for infections?	How many of my patients have a long-term prescription? How many prescriptions were new versus refills?	How many of my patients have a long-term prescription? How many prescriptions were new versus refills?

Micro-level. Claims data of insurers is used to provide feedback on the quality of prescribing to GPs in a report called 'practice mirrors' introduced in 2018. These feedback reports detail the volume and costs of prescriptions and can signal GPs that overuse or underuse prescription medications. GPs participating to the Nivel, Pharmo or academic GP research network datasets receive additional feedback on their prescribing patterns.

Nearly all GPs in the Netherlands participate in pharmacotherapy audit groups (FTOs). FTOs are organised locally and are a practical mechanism for creating linkages between GPs and the pharmacists. As one informant described:

> From my experience as a GP, the FTO is a great mechanism for linking up the GP and the pharmacists as the pharmacist really is the one that has a lot of data on what medicines are being handed out. The pharmacist has a really powerful dataset but they do miss the facts about the patient's actual needs. The linkage [exchange] between a GP and the pharmacists data set happens only at the meeting [FTO] itself. (Health Professional–2)

Informants described the indicators reported at the micro-level vary for reasons primarily due to the type of data available to stakeholders, the priorities of practices and the relevance of existing indicators. On the latter, informants noted differences between feedback that may be useful for a pharmacist versus a GP. For example, from the perspective of pharmacists, the following was described regarding benzodiazepines over an extended period of time:

> There are some indicators to give feedback to pharmacists about whether they give long-term prescriptions to elderly people. But we do not use this as a quality indicator because the pharmacist's care is just a small amount of the care that is provided to patients using benzodiazepines…It depends [rather on] the work of the GPs. (Association–1)

In contrast, from the perspective of GPs, informants described structured feedback on antibiotics as limited by gaps in information, such as the absence of data on how long a patient actually took antibiotics.

Meso-level. Two main types of arrangements are in place for providing feedback at the meso-level. These include regional groups, specifically care groups, as geographically defined networks of

For peer review only - http://bmjopen.bmj.com/site/about/guidelines.xhtml
For peer review only

healthcare providers which provide feedback to affiliated practices. Additionally, research and academic GP networks, such as the Nivel primary care database and GP practices organised around academic hospitals, also conduct research on specific indicators of interest to affiliated GPs.

Dutch professional associations for GPs (eg, National Association of GPs, Dutch GP Association) and pharmacists (eg, Royal Dutch Society for the Promotion of Pharmacy) provide feedback on prescribing for professional development purposes. In the sphere of community pharmacists, the number of medication reviews, participation in pharmacotherapy meetings (FTOs), as well as indicators related to dispensing amounts are regularly measured.

Uses of primary care prescribing data for monitoring purposes by meso-level organisations was described to typically include volume indicators related to the total prescriptions annually, compared to previous years and by age groups. Active monitoring of benzodiazepines at the meso-level was noted to have decreased following changes in reimbursement coverage from January 2009. As one informant explained:

> Around three quarters of prescriptions for benzodiazepines are not reimbursed and data [used] relies on the reimbursement claims. (Association–8)

Moreover, as another informant described with regards to monitoring the uses of prescribing data more locally (e.g., by regions), overall activity is currently limited.

> The discussion on the use of prescriptions at the moment is taking place at the national-level and at the local level but not at the regional-level. This may and is likely to change in the coming years as care groups are more actively involved in the regional implementation of policies. (Association–15).

Macro-level. At the macro-level, pharmacy and claims data are used for strategy development, system performance measurement and quality assurance purposes. Indicators related to the tracer prescriptions are also reported for international comparisons (eg, total volume of antibiotics for systemic use, elderly patients with prescription of long-term benzodiazepines or related drugs and overall volume of opioids prescribed). A number of policy initiatives are in place to monitor antibiotic prescribing and opioids. However, with regards to benzodiazepines, informants described this as a less pertinent priority following the change in reimbursement resulting in an overall decreasing trend in the number of benzodiazepines prescribed.

Optimising the use of primary care prescribing data

Five main themes were identified as areas for optimising the use of primary care prescribing data: (1) measuring what matters, (2) increasing data linkages, (3) improving data quality, (4) facilitating data sharing, and (5) optimising fit for use analysis. Theme one pertains to methodological considerations about the indicators in use, while themes two, three and four relate to contextual considerations, specifically, the underlying information system and regulations. The last theme is found to reflect managerial considerations influencing an indicator's use in practice. The themes are described to follow.

Measuring what matters. *"*We have the data. We don't have the right indicator" (Health professional–2). Similar statements were made in reference to indicators currently in use, in particular at the micro-level. Specifically, the absence of indicators to monitor the stop date of prescriptions were noted, despite the relevance of this information to limit over–re-prescriptions. Information on the stop date for prescriptions was described of growing importance. Notably, as GPs increasingly work in teams and multiple practices, there is greater potential for re-prescribing to go unnoticed. Similarly, the absence of indicators that distinguish between new versus repeat refills, as well as indicators for monitoring "de-prescribing" were noted as an information gap, especially for measuring quality of chronic care services.

The lack of indicators to measure the appropriateness of prescriptions was also raised:

For peer review only - http://bmjopen.bmj.com/site/about/guidelines.xhtml

> Instead of receiving, 'this month you prescribed this many antibiotics' to know 'this month you prescribed this many antibiotics for this many patients diagnosed with infections' can provide more insights into a GP's actual performance. (Association–15)

> Dispensing data we have is really useful for the overall consumption, but it is limited to assess the quality of care. For example, for antibiotics use and to determine the appropriateness of the use you really need to have the diagnosis data. (Association–1)

Increasing data linkages. The interoperability of data systems was a recurrent theme across informants from all levels of the healthcare system. The challenge to link data sources was described both *within* primary care (GPs and community pharmacists) but also *across* levels (GPs, hospitals and community pharmacists). At present, a reliance on manual data exchange between stakeholders was depicted (eg, patients providing data to community pharmacists following hospital discharge, pharmacists providing data to GPs at FTO meetings). While in part a consequence of privacy regulations, informants underscored issues of fragmentation and siloed data systems.

> In a perfect world we would have more linkages between the GP databases and that of the pharmacy. Because we know that the systems in the GP practice is lacking some of the information that is available to the pharmacist. Also, what is prescribed in hospital. We need a connection between these systems to create really good indicators. (Association–8)

In the absence of data linkages within primary care as well as specialised care, informants emphasised the implications on the completeness of data and potential to "see the whole picture" (EHR supplier–10).

Improving data quality. The quality of coding is a fundamental challenge to the secondary use of prescribing data. As one informant described:

> If a GP wants to prescribe antibiotics, then they can also change the code, for example, if someone presents with a possible infection and I see they are quite sick, I can code this differently. (Association–15)

Additionally, the poor quality of coding itself was raised:

> In many GP practices at the moment there is simply not enough attention for the quality of the prescription [coding]. GPs are using very old codes [medication codes] in their prescriptions, simply by way of copying their old prescriptions. (EHR supplier–10)

The pertinence of this issue is well-studied (e.g., [44]) and is underscored in projects such as Nivel's formulary-oriented prescribing initiative (Formulariumgericht voorschrijven) [45], where attention is called to improving the quality of GP prescribing.

Facilitating data sharing. Informants raised privacy barriers as a key cause for untapped opportunities to stimulate data sharing across the healthcare system. The European General Data Protection Regulation (GDPR) and national privacy and data ownership policies were referenced as challenges to the sharing and connecting of different sources of data. As one informant described: "It is a political issue of clarifying who is in fact the owner of the data" (Association–14). Informants emphasised the importance of addressing privacy constraints and data sharing in order to allow for more extensive uses.

Actionable analysis. Informants across all levels described limitations regarding the usefulness of analysed data to inform decision-making. Specifically, at the micro-level opportunities to improve the use of comparators were detailed. For example, the current practice of providing an individual GP with feedback on their performance in relation to the national level was described as too aggregate a summary. The consequence, as one informant noted, is a tendency to defer accountability and cite the

For peer review only - http://bmjopen.bmj.com/site/about/guidelines.xhtml

uniqueness of one's practice population as a cause for deviating trends. In another example, an informant described the compromised actionability of feedback:

> Informing 'you are adhering to guidelines in 80% of prescriptions issued' is not helpful to a GP. It leaves unanswered questions, such as, what patients were involved. (Association–8)

Other obstacles described included the ability to discriminate performances to capture practice variation, with one informant stating: "the problem with the analysis is that the results are not wide. Everyone ends up at the same place" (Insurer–19). Additionally, analysed data fails to capture at-risk patients and vulnerable groups, of relevance across micro-meso-macro contexts. As one informant described from the perspective of pharmacists, current indicators and approaches to analyse information are strained to provide a clear direction for improvement related to care for patients with greatest needs:

> I think we need more data to better target the patients that are in need of additional care. Not everyone needs additional, specialised care. It's the 20% that needs additional, specialized care, and for that, our pharmaceutical database is not sufficient. (Association–1)

Obstacles to analyse data that meets the timeliness needs of decision-makers were also described as a hurdle to the optimal use of data. One informant detailed this challenge extends to the timeliness and accessibility of how data is ultimately delivered to end-users: "We miss a dashboard or system that would allow gaining access and make use of the available data" (Association–12).

For peer review only - http://bmjopen.bmj.com/site/about/guidelines.xhtml

Discussion

In this study, we set out to investigate sources, secondary uses, and key considerations for optimising primary care prescribing data and its actionability for quality of care related decision-making. Much of the existing literature on measurement for improving primary care prescribing focuses on implementation sciences and practice-level interventions (eg, [46-48]). There is also a dedicated field of research on improving prescribing through interventions in direct patient care (eg [27-29]). We add to this evidence by adopting a healthcare performance intelligence lens and exploring the secondary uses of primary care prescribing data for learning and improvement in the Dutch healthcare system.

Our study confirms the numerous secondary uses of electronic primary care data across the clinical, organisational and policy context of the healthcare system in the Netherlands. Nonetheless, data are constrained by professional and organisational siloes and perceived privacy constraints that compromise the completeness of information for secondary uses. Importantly, resolving data-related barriers alone will not increase the use of prescribing data. In addition, attention to the development of strategic, purpose-driven indicators and their embedding in systems of governance and managerial cycles, is needed. These findings are further described to follow.

First, with regards data sources, the incompleteness of individual primary care prescribing data sources is a known limitation [49,50]. Our findings regarding challenges to link available data sources are consistent with recent reporting on the Dutch health information system in general [17] and ultimately, common to many European routine healthcare information systems [50-52]. Importantly, while often justified as a legal constraint, regulations like GDPR in fact leave much room for national legislation [53]. Recent Dutch initiatives like the “Electronic Data Exchange in Health Care Bill” [54] and national quality and information standards for the exchange of medication data [55,56] are important steps being taken for more integrated data at the point of care. However, the same level of policy attention remains needed to ensure that complete data is available for secondary uses.

Second, our findings suggest existing indicators require further development by prescription type and their intended uses. A general fixation on the scientific merits of an indicator in the field of performance measurement has put attention to the development and selection of indicators based on their validity and reliability [57]. However, we observe this focus on scientifically strong indicators in the context of primary care prescribing has distracted from the selection of prescribing indicators based on strategic measurement goals. Our finding that indicators are not differentiated by individual prescription types and information needs of stakeholders attests to this. Similar to previous studies (e.g. [13,58,59]), informants described differences in their desired type of information. The development of indicators with a focus on the *use* and *users* of prescribing indicators to achieve performance goals is needed across the micro-meso-macro level.

Third, putting data to work requires an enabling institutional environment [60]. Realizing learning and improvement in practice across the healthcare system is a matter of good governance and management. Challenges to use primary care prescribing data underscores that the use of indicators is a process. The effective use of indicators relies also on governance considerations such as the mandates of stakeholders and alignment of resources [61]. In the absence of an enabling governance system spanning all levels of the healthcare system [62-64], policy priorities like managing antibiotic resistance and responding to the opioid epidemic, risk to remain solely high-level goals rather than cascading the system. Other governance and managerial considerations include how that information is returned to end-users, such as in reports or dashboards, and ultimately, processes for reflection on the information, need to be fostered and tailored to different stakeholders.

Lastly, we note that despite the range of stakeholders and activities found at each level of the healthcare system, we observe that the current uses of prescribing data are primarily for internal, provider-oriented purposes rather than for public reporting and accountability. However, the prescribing data available has a range of potential uses for the public. These uses include for accountability purposes but also for learning regarding side effects and harms related to the inappropriate use of antibiotics or longer-term

For peer review only - http://bmjopen.bmj.com/site/about/guidelines.xhtml

use of opioids and benzodiazepines, and ultimately, have an important role to play in the patient safety agenda.

Strengths and limitations

This study was enriched by the diverse engagement of stakeholders across all levels of the Dutch healthcare system, resulting in a thorough qualitative dataset. The advanced digitalisation and secondary uses of primary care data in the Dutch setting may be transferable to other data-rich contexts while also serving as an aspirational example for those at an earlier stage of development. For the purposes of this study and its scope, we focused on the use of indicators for antibiotics, benzodiazepines and opioids and the results, therefore, may not reflect the nuances of all prescription types. Other types of medications, such as for chronic conditions, were excluded as the management of healthcare needs is multifaceted and the appropriate rate of prescriptions is highly patient, disease and risk-factor specific. All interviews took place in English with native Dutch-speakers. Lastly, the study by design is exploratory in nature. Therefore, patterns and experiences by stakeholder and data types require testing with a larger sample before they can be generalised. Relatedly, the study has put focus on the secondary uses of prescribing data and, therefore, may not be generalisable to uses for direct patient care, such as in shared decision-making and patient education.

Conclusions

Drawing on the expertise of the diverse sample of stakeholders interviewed, we described the information potential of electronic clinical, administrative, and claims prescribing data for secondary quality of care-related uses. Informants stressed the unique strengths and limitations of available data sources, with the incompleteness of each individually a key challenge. While primary care prescribing data is in use across the Dutch healthcare system, existing indicators require further development. In the case of antibiotics, this is found as a need to better indicate the appropriateness of prescriptions and for benzodiazepines and opioids, to monitoring their long-term use. Beyond methodological considerations about the indicators themselves, contextual considerations related to the information system and regulations as well as managerial considerations influencing an indicator's use in practice are areas identified for further prioritisation. To curb societal concerns like antibiotic resistance and the misuse of opioids and benzodiazepines, the availability of prescribing data alone is insufficient. Available data sources must be linked and made actionable through fit for purpose and fit for use indicators applied at all levels of the healthcare system.

For peer review only - http://bmjopen.bmj.com/site/about/guidelines.xhtml

Contributions

EB, RV, LR, NK and DK conceptualised the study. EB with the support of RV and LR conducted data collection. EB prepared the manuscript. All authors provided feedback and contributed to revising the manuscript. All authors approved the final version.

Ethics and other permissions

The research adheres to the Dutch ethics guidelines stated in the 'Medical Research Act with People (Wet medisch-wetenschappelijk onderzoek met mensen (WMO)) (Dutch), in BWBR0009408, W.a.S. Ministry of Health, Editor. 1998: Hague, Netherlands',51) for which exception applies as no human data were retained and voluntary informed consent of participants was deemed adequate by the authors.

Funding

This work was carried out by the Marie Skłodowska-Curie Innovative Training Network for Healthcare Performance Intelligence Professionals (HealthPros) that has received funding from the European Union's Horizon 2020 research and innovation programme under grant agreement Nr. 765141.

Conflicts of interest

None declared.

Acknowledgements

We thank all participants who provided their time and expertise to this study as well as the time of the Data Expert Community members and reviews by HealthPros fellows, Karin Hek, and other Nivel colleagues. We also thank the reviewers for their thoughtful and constructive feedback.

Data sharing

The dataset supporting the conclusions of this study are included within the article and its supplementary files.

For peer review only

For peer review only - http://bmjopen.bmj.com/site/about/guidelines.xhtml

References

1. OECD. Stemming the Superbug Tide2018.
2. World Health Assembly. Global action plan on antimicrobial resistance. World Health Organization; 2015.
3. United Nations Office on Drugs and Crime. World Drug Report 2019. Vienna, Austria: United Nations 2019. Contract No.: Booklet 1.
4. OECD. Addressing Problematic Opioid Use in OECD Countries2019.
5. Palinkas LA. Opioid Use Epidemic in Mexico: Global Solutions to a Global Problem. American journal of public health. 2019;109(1):26-7.
6. Hayhoe B, Lee-Davey J. Tackling benzodiazepine misuse. BMJ (Clinical research ed). 2018;362:k3208.
7. OECD. Health at a Glance 20192019.
8. OECD. Safe prescribing in primary care. Health at a Glance 2021: OECD Indicators. Paris: OECD; 2021.
9. Bach P, Hartung D. Leveraging the role of community pharmacists in the prevention, surveillance, and treatment of opioid use disorders. Addiction Science & Clinical Practice. 2019;14(1):30.
10. Smith P, Mossialos E, Papanicolas I. Performance measurement for health system improvement: eperiences, challenges and prospects. Copenhagen: WHO Regional Office for Europe; 2008.
11. Mainz J. Defining and classifying clinical indicators for quality improvement. International Journal for Quality in Health Care. 2003;15(6):523-30.
12. Raleigh VS, Foot C. Getting the measure of quality: opportunities and challenges. London: The King's Fund; 2010.
13. Smith P, Mossialos E, Papanicolas I, Leatherman S. Part 1: Principles of performance measurement. In: Smith P, Mossialos E, Papanicolas I, Leatherman S, editors. Performance measurement for health system improvement: experiences, challenges and prospects Copenahgen WHO Regional Office for Europe 2008.
14. van den Broek d'Obrenan J, Verheij TJM, Numans ME, van der Velden AW. Antibiotic use in Dutch primary care: relation between diagnosis, consultation and treatment. Journal of Antimicrobial Chemotherapy. 2014;69(6):1701-7.
15. Verheij RA, van der Zee J. Collecting information in general practice: 'just by pressing a single button'? the Netherlands: NIVEL; 2006.
16. Kroneman M, Boerma W, van den Berg MJ, Groenewegen P, de Jong J, van Ginneken E. The Netherlands: health system review2016. 1–239 p.
17. OECD. Toward an integrated health information system in the Netherlands: Draft interim brief and recommendations. Paris: OECD; 2021.
18. Panch T, Pearson-Stuttard J, Greaves F, Atun R. Artificial intelligence: opportunities and risks for public health. The Lancet Digital Health. 2019;1(1):e13-e4.
19. Verheij RA, Curcin V, Delaney B, McGilchrist MM. Possible sources of bias in primary care electronic health record data use and reuse Journal of medical internet research. 2018;20(5).
20. Barbazza E, Klazinga N, Kringos D. Exploring the actionability of healthcare performance indicators for quality of care: a qualitative analysis of the literature, expert opinion and user experience BMJ Quality and Safety. 2021.
21. Menear M, Blanchette M-A, Demers-Payette O, Roy D. A framework for value-creating learning health systems. Health research policy and systems. 2019;17(1):79.
22. Friedman C, Rubin J, Brown J, Buntin M, Corn M, Etheredge L, et al. Toward a science of learning systems: a research agenda for the high-functioning Learning Health System. J Am Med Inform Assoc. 2015;22(1):43-50.
23. van der Bij S, de Hoon S, Nielen M, de Jong A, de Boer D, Verheij R. Routine recorded care data as a source of quality information [Dutch]. Netherlands Nivel; 2016.
24. Botje D, ten Asbroek G, Plochg T, Anema H, Kringos DS, Fischer C, et al. Are performance indicators used for hospital quality management: a qualitative interview study amongst health

For peer review only

For peer review only - http://bmjopen.bmj.com/site/about/guidelines.xhtml

professionals and quality managers in The Netherlands. BMC Health Services Research. 2016;16(1):574.
25. Giesen P, Willekens M, Mokkink H, Braspenning J, Van Den Bosch W, Grol R. Out-of-hours primary care: development of indicators for prescribing and referring. International journal for quality in health care : journal of the International Society for Quality in Health Care. 2007;19(5):289-95.
26. Bos V, Klazinga NS, Kringos DS. Improving performance intelligence for governing an integrated health and social care delivery network: a case study on the Amsterdam Noord district. BMC Health Serv Res. 2021;21(1):517.
27. Moreira L. Health literacy for people-centred care. 2018.
28. van Esch TEM, Brabers AEM, Hek K, van Dijk L, Verheij RA, de Jong JD. Does shared decision-making reduce antibiotic prescribing in primary care? J Antimicrob Chemother. 2018;73(11):3199-205.
29. Kuntz JL, Safford MM, Singh JA, Phansalkar S, Slight SP, Her QL, et al. Patient-centered interventions to improve medication management and adherence: a qualitative review of research findings. Patient Educ Couns. 2014;97(3):310-26.
30. RIVM. NethMap 2019: Consumption of antimicrobial agents and antimicrobial resistance among medically important bacteria in the Netherlands. RIVM; 2019.
31. Trimbos Institute. National Drug Monitor: Annual Report Utrecht: Trimbos Institute; 2019.
32. Rendle KA, Abramson CM, Garrett SB, Halley MC, Dohan D. Beyond exploratory: a tailored framework for designing and assessing qualitative health research. BMJ open. 2019;9(8):e030123-e.
33. Tong A, Sainsbury P, Craig J. Consolidated criteria for reporting qualitative research (COREQ): a 32-item checklist for interviews and focus groups. International Journal for Quality in Health Care. 2007;19(6):349-57.
34. Vogl S, Schmidt E, Zartler U. Triangulating perspectives: ontology and epistemology in the analysis of qualitative multiple perspective interviews. International Journal of Social Research Methodology. 2019;22(6):611-24.
35. Schweikardt C, Verheij RA, Donker GA, Coppieters Y. The historical development of the Dutch Sentinel General Practice Netowrk from a paper-based into a digital primary care monitoring system J Public Health. 2016.
36. Verheij R, Witvliet C, Jansen T, Hooiveld M, Hilten O van. Reuse of routine care data for policy and science: how things can be improved [Dutch]. Utrecht: Nivel; 2019.
37. Braun V, Clarke V. Using thematic analysis in psychology. Qualitative Research in Psychology. 2006;3(2):77-101.
38. Meyer DZ, Avery LM. Excel as a Qualitative Data Analysis Tool. Field Methods. 2008;21(1):91-112.
39. Medical Research Act with People (Wet medisch-wetenschappelijk onderzoek met mensen (WMO)) [Dutch], (1998).
40. Stoker LJ, Heerdink ER, Janssen R, Egberts TCG. Effect of reimbursement restriction policy on the use of benzodiazepines in the Netherlands: an interrupted time series analysis. BMJ Open. 2019;9(9):e029148.
41. NIVEL. Nivel Primary Care Database: NIVEL 2019 [Available from: https://www.nivel.nl/en/nivel-primary-care-database.
42. Kollen BJ, van der Veen WJ, Groenhof F, Donker GA, van der Meer K. Discontinuation of reimbursement of benzodiazepines in the Netherlands: does it make a difference? BMC family practice. 2012;13(1):111.
43. Lareb NPC. About Lareb 2020 [Available from: https://www.lareb.nl/en/pages/about-lareb.
44. Verheij RA, Curcin V, Delaney BC, McGilchrist MM. Possible Sources of Bias in Primary Care Electronic Health Record Data Use and Reuse. J Med Internet Res. 2018;20(5):e185.
45. Ramerman L, Hek K, Sluiter R, Middelweerd A, Meijer A, Dijk L, et al. Formulary-oriented prescribing by the general practitioner: instrument development and monitoring 2018. Utrecht: Nivel; 2020.
46. Trietsch J, van Steenkiste B, Grol R, Winkens B, Ulenkate H, Metsemakers J, et al. Effect of audit and feedback with peer review on general practitioners' prescribing and test ordering performance: a cluster-randomized controlled trial. BMC family practice. 2017;18(1):53.

For peer review only - http://bmjopen.bmj.com/site/about/guidelines.xhtml

47. Rowe TA, Linder JA. Novel approaches to decrease inappropriate ambulatory antibiotic use. Expert Rev Anti Infect Ther. 2019;17(7):511-21.
48. van Eeghen C, Kennedy AG, Pasanen ME, MacLean CD. A New Quality Improvement Toolkit to Improve Opioid Prescribing in Primary Care. The Journal of the American Board of Family Medicine. 2020;33(1):17.
49. Williams R, Ashcroft DM, Brown B, Rojas E, Peek N, Johnson O. Process Mining in Primary Care: Avoiding Adverse Events Due to Hazardous Prescribing. Studies in health technology and informatics. 2019;264:447-51.
50. Okoli GN, Myles P, Murray-Thomas T, Shepherd H, Wong ICK, Edwards D. Use of Primary Care Data in Research and Pharmacovigilance: Eight Scenarios Where Prescription Data are Absent. Drug Safety. 2021.
51. Saigí-Rubió F, Pereyra-Rodríguez JJ, Torrent-Sellens J, Eguia H, Azzopardi-Muscat N, Novillo-Ortiz D. Routine Health Information Systems in the European Context: A Systematic Review of Systematic Reviews. International Journal of Environmental Research and Public Health. 2021;18(9).
52. Bogaert P, Verschuuren M, Van Oyen H, van Oers H. Identifying common enablers and barriers in European health information systems. Health Policy. 2021.
53. Hansen J, Wilson P, Verhoeven E, Kroneman M, Kirwan M, Verheij R, et al. Assessment of the EU Member States' rules on health data in light of GDPR. Brussels: European Commission; 2021.
54. Rules regarding the electronic sharing and access of data between healthcare providers in designated data exchanges (Electronic Data Exchange in Healthcare Act), (2021).
55. Directive Transfer of Medication data in the chain Review 2018/2019, (2019).
56. Nictiz. Information standard: digital recipe traffic NEN7503 2021 [Available from: https://www.nictiz.nl/standaardisatie/informatiestandaarden/medicatieveiligheid/Informatiestandaard/#functioneel.
57. Loeb JM. The current state of performance measurement in health care. International journal for quality in health care : journal of the International Society for Quality in Health Care. 2004;16 Suppl 1:i5-9.
58. Damberg CL, Sorbero ME, Lovejoy SL, Lauderdale K, Wertheimer S, Smith A, et al. An Evaluation of the Use of Performance Measures in Health Care. Rand health quarterly. 2012;1(4):3-.
59. Palin V, Tempest E, Mistry C, van Staa TP. Developing the infrastructure to support the optimisation of antibiotic prescribing using the learning healthcare system to improve healthcare services in the provision of primary care in England. BMJ Health Care Inform. 2020;27(1).
60. OECD. Health in the 21st Century: putting data to work for stronger health systems. Paris: OECD; 2019.
61. Barbazza E, Tello J. A review of health governance: Definitions, dimensions and tools to govern Health Policy 2014;116:1-11.
62. Jeremy Veillard THSASKNSK, Adalsteinn DB. Making Health System Performance Measurement Useful to Policy Makers: Aligning Strategies, Measurement and Local Health System Accountability in Ontario. Healthcare Policy. 2010;5(3):49-65.
63. Travis P, Egger D, Davies P, Mechbal A. Towards better stewardship: concepts and critical issues. Geneva: World Health Organization; 2002.
64. Baez-Camargo C. Accountability for better healthcare provision: A framework and guidelines to define, understand and assess accountability in health systems. Basel: Institute on Governance; 2011.

For peer review only - http://bmjopen.bmj.com/site/about/guidelines.xhtml

Optimizing the secondary use of primary care prescribing data to improve quality of care: a qualitative analysis

Supplementary file 1: Interview topic guide

1. Purpose of use of primary care prescribing data

- How does your organization currently use primary care prescribing data? Refer to the table on the following page (Supplementary file 2) listing core actors and purposes of use identified. Is this accurate and complete?

- How would you describe the information you need to carry-out your organization's role? (e.g. multi-year information on performance at national-level; aggregate, comparative performance measures on providers; timely, continuous information at patient-level, etc.).

- What actors do you work with directly in the scope of primary care prescribing data?

2. Current use of prescribing data

- **Use of indicators**. Does your organization actively collect data related to the following: (1) Antibiotics; (2) Opioids; (3) Benzodiazepines. If so, what are the indicators or measures used related to each? How long have these been reported on? Is it intended for internal or external use? Who is the target audience (intended user) of the information generated?

- **Data sources**. What is your primary source of primary care prescribing data? (e.g. medical records, administrative data, specific research database, others). How is the data collected? If other actors are involved, whom does this include? Is the data considered of quality?

- **Analysis.** How is the data currently analyzed – benchmarking, time trend, international comparison? How would you describe this analysis? (e.g. time interval, comparators used, aggregation as composite scores, etc.)

- **Dissemination**. How is the data disseminated? What is the format of reporting (print, electronic, web-based)? What is the lag time in presenting analyzed data? How does it reach the intended target audience?

3. Perceived actionability

- In your opinion, how can the process in which data is analyzed and reported on be improved upon?

- Is the information generated useful for your purposes? That is, are you able to make decisions and learn from the information?

- In general, what are the obstacles to the optimal use of primary care prescribing data at present?

For peer review only - http://bmjopen.bmj.com/site/about/guidelines.xhtml

Supplementary file 2: Mapping of stakeholders and uses of prescribing data

Purpose of use[1]	Stakeholders
**Micro-level**	
Individual professional performance	Individual GP and HIS supplier<br>Community pharmacist and HIS supplier
Practice improvement	GP practice/peers using HIS<br>GP practice and insurers<br>GP practice and affiliate research networks<br>Community pharmacy and pharmacy network
Multidisciplinary improvement	Pharmacotherapy audit groups (GPs and pharmacists) (FTOs)
**Meso-level**	
Organization/ networks performance improvement	Care groups and affiliate GP practices (e.g., MCC Omens Care Group, Zorg In Ontwikkeling)
Quality-based financing	Health Insurers (e.g., Zilveren Kruis)
Monitoring	Lareb Side Effects Center
Professional development	Foundation of Pharmaceutical Statistics (SFK)<br>Dutch Institute for Responsible Drug Use (IVM)<br>National Association of GPs (LHV)
Advocacy and standards	Dutch GP Association (NHG)<br>The Royal Dutch Society for the Promotion of Pharmacy (KNMP)<br>Organization for first line care (InEen)<br>Patient Federation Netherlands (Patienten Federatie)
**Macro-level**	
Strategy development	Ministry of Health, Welfare and Sport
System performance	National Institute for Public Health and the Environment (RIVM)
System quality assurance	Medicines Evaluation Board (MEB)<br>National Health Care Institute (ZiNL)<br>Health Care Inspectorate (IGJ)<br>Dutch Healthcare Authority (NZa)
**Cross-cutting**	
	Netherlands Institute for Health Services Research (Nivel)<br>Institute for Drug Outcomes Research (Pharmo)<br>Vektis<br>Nictiz<br>Digitalis

[1]Purposes of use draw from the study findings: Barbazza E, Klazinga NS, Kringos DS. Exploring the actionability of healthcare performance indicators for quality of care: a qualitative analysis of the literature, expert opinion and user experience. BMJ Quality & Safety 2021;30:1010-1020.

For peer review only - http://bmjopen.bmj.com/site/about/guidelines.xhtml

Supplementary file 3: Characteristics of informants

Table S3.1. Elaborated breakdown of informants and non-participants

Characteristics	Total informants N=28		Non-participants N=25		
	**n**	**%**	**No reply**	**Unavailable**	**Contact mediating**
**Healthcare system level (context)**					
Micro (clinical)	1 (4)	4	1	2	1
Meso (organizational)	11	39	6	0	5
Macro (policy)	9	32	3	0	2
Cross-cutting (research, EHR supplier)	7	25	2	1	2
**Type of stakeholder**			**12**	**3**	**10**
Association (patient, professional)	8	29	3	0	2
Care group (network)	2	7	0	1	0
Government health agency	9	32	3	0	2
Health professional	1 (4)	4	0	1	1
EHR supplier	4	14	1	0	2
Insurer	1	4	3	0	3
Research	3	11	2	1	0
**Gender**					
Female	8	29	4	2	4
Male	20	71	8	1	6

EHR: Electronic health record.
[a]Numbers in round brackets indicate the total number of informants when individuals with multiple affiliations are accounted for.

Table S3.2. Overview of informants

#	Code	Level	Stakeholder	Gender	Format
1	Association–1	Meso	The Royal Dutch Society for the Promotion of Pharmacy (KNMP)	Female	Phone
2	Health professional–2	Micro	Health Professional	Male	Phone
3	Care group–3	Meso	MCC Omens Care Group	Female	Phone
4	Care group–4	Meso	ZIO	Female	Phone
5	Government–5	Macro	Medicines Evaluation Board (MEB)	Male	Phone
6	EHR supplier–6	Cross-cutting	Digitalis	Male	In-person
7	Association–7	Meso	Organization of Firstline Care (InEen)	Male	Phone
8	Association–8	Meso	Dutch Institute for Responsible Drug Use (IVM)	Female	Phone
9	EHR supplier–9	Cross-cutting	Nictiz	Male	Written
10	EHR supplier–10	Cross-cutting	CampuGroup Medical (CGM)	Male	Phone
11	Government–11	Macro	Ministry of Health, Welfare and Sport	Male	Phone
12	Association–12	Meso	Lareb Side Effects Center	Male	Phone
13	Association–13	Meso	Foundation for Quality Indicators Pharmacy (SFK)	Male	Phone

For peer review only - http://bmjopen.bmj.com/site/about/guidelines.xhtml

#	Code	Level	Stakeholder	Gender	Format
14	Government–14	Macro	National Institute for Public Health and the Environment (RIVM)	Male	Written
15	Association–15	Meso	Dutch General Practitioners Association (NHG)	Male	Phone
16	Government–16	Macro	Health Care Inspectorate	Male	Phone
17	Research–17	Cross-cutting	Institute for Drug Outcomes Research (Pharmo)	Male	Phone
18	Association–18	Meso	Patient Federation	Male	Phone
19	Insurer–19	Meso	Zilveren Kruis	Male	Phone
20	Association–20	Meso	National General Practitioners Association (LHV)	Male	Phone
21	EHR supplier–21	Cross-cutting	Vektis	Male	Phone
22	Government agency–22	Macro	National Health Care Institute	Male	Phone
23	Government agency–23	Macro	Ministry of Health, Welfare and Sport	Male	Phone
24	Government agency–24	Macro	Dutch Healthcare Authority	Female	Phone
25	Government agency–24	Macro	Dutch Healthcare Authority	Male	Phone
26	Research–25	Cross-cutting	Netherlands Institute for Health Services Research	Female	In-person
27	Research–25	Cross-cutting	Netherlands Institute for Health Services Research	Female	In-person
28	Government agency–26	Macro	National Institute for Public Health and the Environment (RIVM)	Female	Phone

EHR: Electronic health record.

For peer review only

For peer review only - http://bmjopen.bmj.com/site/about/guidelines.xhtml

Optimizing the secondary use of primary care prescribing data to improve quality of care: a qualitative analysis

Consolidated Criteria for Reporting Qualitative Research (COREQ) Checklist[a]

#	Item	Question/description	Answer	Section (page, para)
**Domain 1: Research team and reflexivity**				
**Personal Characteristics**				
1	Interviewer/ facilitator	Which author/s conducted the interview or focus group?	EB	Methods (pg 5, para 1)
2	Credentials	What were the researcher's credentials?	BHSc, MSc, PhD candidate	Methods (pg 5, para 1)
3	Occupation	What was their occupation at the time of the study?	HealthPros Fellow and PhD candidate Department of Public and Occupational Health University of Amsterdam	Methods (pg 5, para 1)
4	Gender	Was the researcher male or female?	Female	Title page
5	Experience and training	What experience or training did the researcher have?	At the time of the interviews, had completed more than five years of interview-based data collection.	Methods (pg 5, para 1)
**Relationship with participants**				
6	Relationship established	Was a relationship established prior to study commencement?	Yes, informants were contacted via email and corresponded with the interviewer to confirm their interest and agreement to participate.	Methods (pg 5, para 4)
7	Participant knowledge of the interviewer	What did the participants know about the researcher? e.g. personal goals, reasons for doing the research.	Participants received a study brief prior to the interview detailing the study's purpose, aims, funding and dissemination of results as well as the questions to be discussed.	Methods (pg 5, para 5), Supplementary file 1
8	Interviewer characteristics	What characteristics were reported about the interviewer/facilitator? e.g. Bias, assumptions, reasons and interests in the research topic	EB acknowledged her role as a research fellow on health care performance intelligence and that the study was being conducted in the scope of her PhD research.	Methods (pg 5, para 5)
**Domain 2: study design**				
**Theoretical framework**				
9	Methodological orientation and Theory	What methodological orientation was stated to underpin the study? e.g. grounded theory, discourse analysis, ethnography,	Exploratory study design using multiple perspective semi-structured interviews and thematic analysis.	Methods (pg 5, para 1)

For peer review only

#	Item	Question/description	Answer	Section (page, para)
		phenomenology, content analysis		
**Participant selection**				
10	Sampling	How were participants selected? e.g. purposive, convenience, consecutive, snowball	Purposive including snowballing and then sampling to sufficiency	Methods (sample and recruitment selection, pg 5 para 3)
11	Method of approach	How were participants approached? e.g. face-to-face, telephone, mail, email	Via email	Methods (pg 5, para 4)
12	Sample size	How many participants were in the study?	28 informants	Results (pg 7, para 1)
13	Non-participation	How many people refused to participate or dropped out? Reasons?	Non-participants (n=25) either were unreachable (n=12), referred to an alternative contact (n=10) that was met with to follow or were unavailable due to time constraints (n=3).	Results (pg 7, para 1)
**Setting**				
14	Setting of data collection	Where was the data collected? e.g. home, clinic, workplace	Interviews took place by telephone and in-person.	Methods (pg 5, para 5)
15	Presence of non-participants	Was anyone else present besides the participants and researchers	No non-participants were present during data collection.	Results (pg 7, para 1)
16	Description of sample	What are the important characteristics of the sample? e.g. demographic data, date	Table 1 profiles the panelists by their uses of primary care prescribing data, affiliate organizations and gender	Results (pg 7, para 1)
**Data collection**				
17	Interview guide	Were questions, prompts, and guides provided by the authors? Was it pilot tested?	Participants received in advance of the interview the key questions in written form.	Methods (pg 5, para 5)
18	Repeat interviews	Were repeat interviews carried out? If yes, how many	No	Results (pg 7, para 1)
19	Audio/visual recording	Did the research use audio or visual recording to collect the data?	Yes, interviews were audio recorded in agreement with informants.	Methods (pg 5, para 5)
20	Field notes	Were field notes made during and/or after the interview or focus group?	No	Methods (pg 5, para 5)
21	Duration	What was the duration of the interviews or focus groups?	Interviews lasted between 30–60 minutes	Methods (pg 5, para 5)
22	Data saturation	Was data saturation discussed?	Yes, data saturation was reached when stakeholders spanning the differentiated uses of primary care prescribing data were met.	Methods (pg 5, para 5)
23	Transcripts returned	Were transcripts returned to participants for comment and/or correction?	No.	Methods (pg 6)
**Domain 3: analysis and findings**				

For peer review only

#	Item	Question/description	Answer	Section (page, para)
**Data analysis**				
24	Number of data coders	How many data coders coded the data?	EB	Methods (pg 5-6)
25	Description of the coding tree	Did authors provide a description of the coding tree?	Yes, the main level codes are reported.	Methods, data analysis (pg 5-6)
26	Derivation of themes	Were themes identified in advance or derived from the data?	Main level codes were derived from an existing classification of actionable healthcare performance indicators. Unrestricted coding was applied to identify new themes.	Methods, data analysis (pg 5-6)
27	Software	What software, if applicable, was used to manage the data?	Microsoft Word and Excel	Methods, data analysis (pg 5-6)
28	Participant checking	Did participants provide feedback on the findings	Results were shared publicly at a conference.	Methods (pg 6)
**Reporting**				
29	Quotations pr-esented	Were participant quotations presented to illustrate the themes/findings? Was each quotation identified? e.g. participant number	Yes, throughout results. Confidentiality was assured by referring to informants by stakeholder type and a randomly assigned number (e.g., Health professional–1).	Results (pg 7-13)
30	Data and findings consistent	Was there consistency between the data presented and the findings?	Findings are presented in the approach of the research questions and interview topic guide.	Results (pg 7-13)
31	Clarity of major themes	Were major themes clearly presented in the findings?	Tables are used to present major themes. Subheadings are used to improve clarity.	Results (pg 7-13)
32	Clarity of minor themes	Is there a description of diverse cases or discussion of minor themes?	Findings are supplemented in-text by quotes and descriptions of sub-themes.	Results (pg 7-13)

[a] Tong A, Sainsbury P, Craig J. Consolidated criteria for reporting qualitative research (COREQ): a 32-item checklist for interviews and focus groups. Int J Qual Health Care 2007;19:349–57.

For peer review only
